# Real-world objects are not stored in holistic representations in visual working memory

**DOI:** 10.1167/jov.21.3.18

**Published:** 2021-03-17

**Authors:** Yuri A. Markov, Igor S. Utochkin, Timothy F. Brady

**Affiliations:** 1 HSE University, Moscow, Russia; 2 Psychology Department, University of California, San Diego, La Jolla, CA, USA

**Keywords:** visual working memory, feature binding, real-world objects

## Abstract

When storing multiple objects in visual working memory, observers sometimes misattribute perceived features to incorrect locations or objects. These misattributions are called binding errors (or swaps) and have been previously demonstrated mostly in simple objects whose features are easy to encode independently and arbitrarily chosen, like colors and orientations. Here, we tested whether similar swaps can occur with real-world objects, where the connection between features is meaningful rather than arbitrary. In Experiments 1 and 2, observers were simultaneously shown four items from two object categories. Within a category, the two exemplars could be presented in either the same or different states (e.g., open/closed; full/empty). After a delay, both exemplars from one of the categories were probed, and participants had to recognize which exemplar went with which state. We found good memory for state information and exemplar information on their own, but a significant memory decrement for exemplar–state combinations, suggesting that binding was difficult for observers and swap errors occurred even for meaningful real-world objects. In Experiment 3, we used the same task, but in one-half of the trials, the locations of the exemplars were swapped at test. We found that there are more errors in general when the locations of exemplars were swapped. We concluded that the internal features of real-world objects are not perfectly bound in working memory, and location updates impair object and feature representations. Overall, we provide evidence that even real-world objects are not stored in an entirely unitized format in working memory.

## Introduction

Working memory is a limited capacity system (Cowan, 2001; Miller, 1956) used to actively maintain and work with the information necessary for our current goals and tasks (Baddeley, 1986; Baddeley & Hitch, 1974). However, it is unclear what the nature is of the representations stored in visual working memory. Most early work suggested that information about entire objects is represented in intrinsically holistic, totally bound units, with all features stored or forgotten together (Cowan, Chen, & Rouder, 2004; Kahneman, Treisman, & Gibbs, 1992; Lee & Chun, 2001; Luck & Vogel, 1997; Luria & Vogel, 2011; Treisman, 1999; Vogel, Woodman, & Luck, 2001; Xu, 2002; Xu & Chun, 2006); however, since those studies were undertaken, many other studies have provided evidence that there is—either also or instead—relatively independent feature storage (see Brady, Konkle, & Alvarez, 2011, for review;; Bays, Catalao, & Husain, 2009; Bays, Wu, & Husain, 2011; Fougnie & Alvarez, 2011; Fougnie, Cormiea, & Alvarez, 2013; Markov, Tiurina, & Utochkin, 2019; Pertzov, Dong, Peich, & Husain, 2012; Shin & Ma, 2017; Wang, Cao, Theeuwes, Olivers, & Wang, 2017; Wheeler & Treisman, 2002).

In the foundational study about this issue, Luck and Vogel (1997) claimed that only objects, not features, limit the capacity of visual working memory, because they found no decrement in performance with additional features per object, even within the same dimension. Luck and Vogel (1997) suggested, therefore, that unlike in perception, where illusory conjunctions occur and features seem to be unbound to some extent (Treisman, 2006), unitized objects are the “units” of visual working memory. This finding was in line with the “strong” object hypothesis, which claims that visual working memory is limited only by a number of objects and that features play no role in working memory limits and are only forgotten when entire objects are forgotten. However, further research provided evidence against this “strong” object view: multiple features from the same dimension cannot be stored without cost, even if they are on the same objects (Olson & Jiang, 2002; Wheeler & Treisman, 2002); detecting changes that require binding is harder than detecting changes that do not (van Lamsweerde, et al. 2015); additional resources are needed for keeping bound features in memory (Fougnie, Asplund, & Marois, 2010; Fougnie & Marois, 2009); features can independently fade from memory (Fougnie & Alvarez, 2011; Fougnie, Cormiea, & Alvarez, 2013); and memories for different sensory dimensions rely on independent storage capacities (Markov et al., 2019; Wang, Cao, Theeuwes, Olivers, & Wang, 2017). Thus, although the early evidence was mixed on this issue, it is now clear that for simple stimuli like colors and orientations, items in working memory are not inherently represented in a solely object-based, unitized manner (e.g., Cowan et al., 2013; Park et al., 2017). Although participants can maintain the binding between features (at least partially by using location as a cue), this is not because the objects are themselves stored in a single holistic representation that by necessity is encoded and forgotten in an all-or-none manner, as suggested by early work; instead, features rely on distinct capacities, accumulate independent noise, and can be lost independently.

One result of this is that, similar to perceptual illusory conjunctions, binding errors also occur in visual working memory studies (Bays, 2016; Bays, Catalao, & Husain, 2009; Bays, Wu, & Husain, 2011; Dent & Smyth, 2005; Emrich & Ferber, 2012; Pertzov et al., 2012), and this occurs even though these studies use displays where such errors are unlikely to have perceptual origins (e.g., for a set of four objects the percentage of binding errors is approximately 10%; see Bays et al., 2009; Emrich & Ferber, 2012). This finding suggests that memory representations are also prone to the binding problem, at least in circumstances where location noise is considerable (Oberauer & Lin, 2017). Thus, the evidence suggests that both the binding of features within an object and the binding to location of objects are often imperfect in visual working memory.

## Are real-world objects likely to be stored holistically in visual working memory?

Existing studies have almost exclusively tested simple objects with features that are easy to manipulate in experiments (geometrical shapes with various colors, orientations, etc.). There is very little research examining the storage of real-world objects in visual working memory. In comparison with the simple objects that are usually used in standard visual working memory tasks, real-world objects have many more features, both visual and semantic. and the connections between these features are meaningful rather than arbitrary. Although simple features (e.g., color and orientation) each have somewhat or complete independence in their underlying storage capacity (Markov et al., 2019; Wang et al., 2017) and are in some cases even represented by separate neurons or structures (Conway, 2009; Paik & Ringach, 2011), the complex features of real-world objects are not nearly as separable from each other as basic visual features like color and orientation, at least in terms of their visual properties: a cabinet being “open”, for example (rather than “closed”), results in changes to spatial frequency, color, orientation, shape, and many other visual aspects of the object. Thus, one possibility is that real-world objects are stored in memory in a way that is effectively unitized—that, rather than distinct features being encoded and lost separately, and requiring effort or resources to bind, the objects are stored and remembered in a wholly all-or-none manner.

There are several bodies of work from outside of visual working memory that are consistent with this possibility and that can be interpreted as predicting that object memories should be holistic and all-or-none. For example, a large body of work looking at ventral visual processing shows that the individual low-level features that make something an object (e.g., a mug)—the curves and colors and spatial frequencies—are untangled during visual processing into a more general mug representation as processing proceeds to higher level visual areas (DiCarlo, Zoccolan, & Rust, 2012). Thus, unlike low-level features with arbitrary bindings, there are preexisting mid- and high-level representations of many aspects of real-world objects that could be used for working memory storage, perhaps suggesting that, if memory relies on such high-level representations, memory should consist of a relatively unitized object representation (e.g., a mug representation), rather than separable memories for separate properties of objects. Indeed, some studies on cortical representation of objects argues in that the medial temporal lobe as well as more ventral visual regions, objects (Erez, Cusack, Kendall, & Barense, 2016) or structured scenes or events (e.g., van den Honert, McCarthy & Johnson, 2017) are represented holistically; that is, that brain responses cannot be explained by the sum of the component stimuli or features alone (Erez et al., 2016). This hypothesis has been used to argue that a central feature of building more complex object and scene representations—and holding them in memory—is a holistic representation (e.g., van den Honert et al., 2017) that does not rely simply on the similarity of the underlying feature representations but goes beyond these to novel, unitized representations designed to prevent confusions of similar items.

In addition to the question of binding, there are also other pieces of evidence consistent with the idea that participants store real-world objects differently than simpler objects, which could result in qualitatively distinct representations. For example, real-world objects, compared with simple stimuli, allow access to significant additional information (e.g., the real size of the objects: Konkle & Oliva, 2012; Long, Konkle, Cohen, & Alvarez, 2016; Long, Moher, Carey, & Konkle, 2019; expected nearby objects and their spatial position: Kaiser, Stein, & Peelen, 2015); O’Donnell, Clement, & Blockmole, 2018, and people can have specific expertise with certain object categories (Curby & Gauthier, 2007; Curby, Glazek, & Gauthier, 2009; Janini & Konkle, 2019; Xie & Zhang, 2017), all of which may be used to enhance working memory. In fact, several studies have shown that the capacity of visual working memory for real-world objects differs from that of simple stimuli, in particular being less fixed and more dependent on the particular stimuli used and how much meaningful information about them can be processed (Asp, Störmer & Brady, 2019; Brady, Störmer, & Alvarez, 2016). For example, Brady et al. (2016) showed a boost in performance for real-world objects that was attributable to more active storage in visual working memory, consistent with a theory where additional high-level information about such objects, perhaps in the ventral stream, is maintained in working memory in addition to low-level information. Some recent studies (Li, Xiong, Theeuwes, & Wang, 2020; Quirk, Adam, & Vogel, 2020) instead found no difference between storing simple features and real-world objects in visual working memory, but these results were likely due to a lack of control for similarity between targets and foils in the color versus real-world object tasks (Brady & Störmer, 2020; Brady & Störmer, in press). With better control for target–foil similarity (Brady & Störmer, 2020), real-world objects result in significantly better performance compared with simple features (Brady & Störmer, 2020; Brady & Störmer, in press).

Altogether, then, there is significant evidence that real-world objects differ from simple stimuli in working memory and there are reasons to believe that real-world objects may be stored in a more holistic manner because they depend on more high-level representations that have been argued to be based on unitized object representations. Are real-world objects, then, stored in a unitized, all-or-none format in visual working memory? Or can different features of such objects be lost independently, or misbound?

This subject has been largely unaddressed to date. In fact, there are only a few studies that have used real-world objects to investigate binding in visual working memory, and this work has been done mainly in the context of object–location binding (e.g., Lew & Vul, 2015; Pertzov et al., 2012). In such tasks, researchers test memory for item identities and for their locations. In such object–location tests, memory failures can come from forgetting objects, forgetting locations, or forgetting which object was in which location. Because it is widely believed that objects and locations are stored relatively independently, even for realistic objects (e.g., ventral and dorsal pathways; Haxby et al., 1991; Mishkin & Ungerleider, 1982), it is perhaps not surprising that such independence has been found in such studies. However, whether the object's internal features are themselves stored in a holistic, bound representation is not considered within the scope of this existing object–location binding research.

The question of whether real-world objects are stored in bound units has, however, been previously addressed in the studies of visual long-term memory (Balaban, Assaf, Arad Meir, & Luria, 2020; Brady, Konkle, Alvarez, & Oliva, 2013; Spachtholz & Kuhbandner, 2017; Utochkin & Brady, 2020). These studies have largely found evidence supporting a “weak” object view; what Olson and Jiang (2002) used to designate representations that are somewhat bound—that is, people know which features went with which others to some extent—but that are not intrinsically all-or-none representations, and certainly not totally holistic representations (as in Luck & Vogel, 1997).

**Figure 1.**
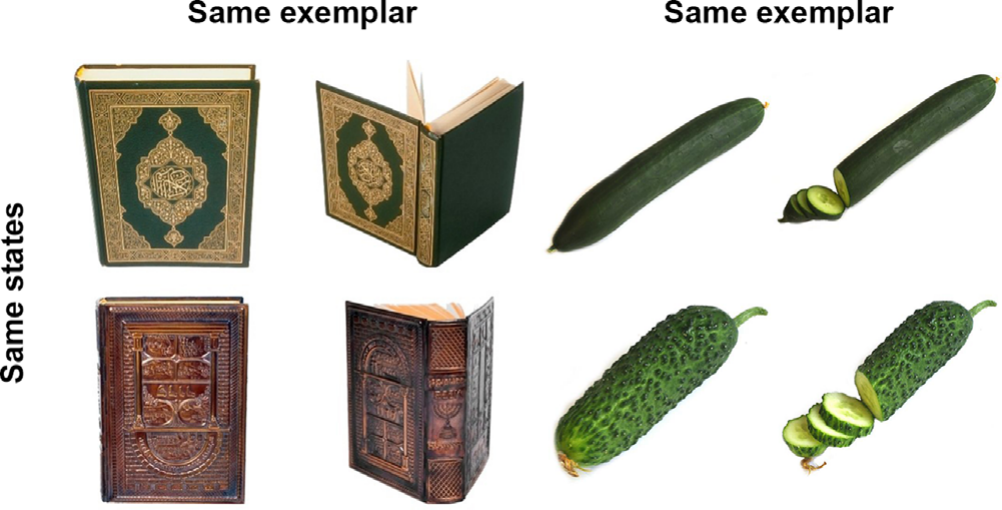
Example of two different exemplars in two different states.

For example, Brady et al. (2013) showed that information about an object's color is forgotten faster than information about the state the object was in (whether the cucumber was whole or sliced; whether the book was open or closed; Figure 1). Furthermore, they showed forgetting is independent for information about object state and information about the specific object exemplar (whether I saw this or that cucumber; or this book or that one; see Figure 1). In other words, Brady et al. showed that the features of real-world objects can be forgotten independently. This finding is consistent with the demonstrations of independent forgetting of simple features in working memory (Fougnie & Alvarez, 2011; Fougnie, Cormiea, & Alvarez, 2013), and suggestive that the representation of real-world objects, at least in long-term memory, is higher level; that is, that information about state and exemplar are encoded distinctly. In their recent work, Utochkin and Brady (2020) tested other predictions that derive from the idea of independent storage of real-world object features in long-term memory. In particular, they demonstrated that people often commit “swap” errors, when they show good memory for both which exemplars and which object states they have seen, but frequently choose an incorrect combination of the exemplar and the state. In particular, Utochkin and Brady (2020) asked participants to remember different exemplars from the same category presented in the same state (e.g., two different coffee mugs, both empty) or in different states (e.g., two different coffee mugs, one empty and one full). They found that participants had good memories for the states and exemplars alone, but when the two exemplars had been seen in different states, participants were at chance in correctly matching which state went with which exemplar, often reporting swapped states for the two exemplars. Thus, Utochkin and Brady (2020) concluded that state and exemplar information are represented independently, rather than in an all-or-none, holistic representation in long-term memory—rejecting the “strong” object claim and casting doubt on even “weak” object-based representations in favor of largely independent storage of high-level object properties like exemplar and state.

Although this work argues for extremely independent representations, other work provides evidence in favor of at least a “weak” object-based view, that is, representations that are dependent between features, even if not all-or-none or holistic. For example, Balaban et al. (2020) argued that there is a dependence between different features of objects in long-term memory, and that at minimum state information cannot be stored without storing the exemplar information, so that exemplars and states are stored in a hierarchical structure or some other form of dependence between them is present. However, the hierarchical structure is not consistent with strong, holistic all-or-none object-based views and leaves the nature of the dependence unclear.

This evidence of at least partially independent long-term memory storage of different features of real-world objects leads to important questions about the representation of these objects in working memory. Are high-level properties of these objects (like object state vs. object exemplar) more holistic and integrated when stored in working memory, which may rely more on sustained perceptual activation in high-level visual areas than does long-term memory? Many accounts of working memory suggest that working memory is more focused on the storage of perceptual features per se, rather than semantic features, suggesting that items are stored in a more perceptual format in working memory than long-term memory (e.g., Baddeley, 1966). As applied to visual working memory, this account would suggest that even real-world objects might be stored solely as perceptual features. This account would be broadly consistent with models of visual working memory that argue memory storage occurs sustained activity in sensory visual cortex; that is, even real-world objects are stored in terms of their colors and shapes and orientations, not in terms of semantically meaningful object features (e.g., Harrison & Tong, 2009; Serences, Ester, Vogel, & Awh, 2009; for review, see Serences, 2016). If this is the case—that visual working memory is strictly perceptual—then nearly all high-level changes to real-world objects (like a change of the state or exemplar) would be expected to appear strongly bound in this memory system, because almost any change in either dimension would change the color, shape, orientation, and so on. By contrast, because distinguishing between two different states or poses of an object, and between two different exemplars of the same object category are two common and important tasks that we perform every day in the context of real-world objects, if objects are stored in more meaningful representations, rather than purely as perceptual features, they could appear quite independent. Indeed, this would be consistent with some evidence that the inferior temporal cortex, where objects are likely to be represented in richer ways (e.g., via parts) is involved in visual working memory (Fiebach, Rissman, & D'Esposito, 2006; Li, Miller, & Desimone, 1993; Miller, Erickson, & Desimone, 1996; Ranganath, DeGutis, & D'Esposito, 2004), and with suggestions that visual working memory involves more abstracted representations in parietal cortex rather than low-level sensory representations (e.g., Xu, 2017). Thus, to some extent the question of how bound they are in working memory is a question about the nature of visual working memory storage for real-world objects more generally.

In addition, if we consider working memory representation as a precursor for consolidation into long-term memory, then it could be that the independent long-term memory storage of such high-level features like object state and object exemplar could plausibly be accounted for by limited binding in working memory. Utochkin and Brady (2020) briefly addressed this issue using a short-term memory version of their exemplar–state memory task. However, their task was extremely straightforward, with only two objects to remember, and thus did not load working memory capacity in the ways that are known to cause more misbinding reports for simple stimuli and basic features (Bays et al., 2009, 2011; Emrich & Ferber, 2012; Pertzov et al., 2012).

Thus, in the current study, we tested the boundedness of real-world object representations in visual working memory using a relatively high-load task requiring participants to remember four items presented simultaneously, akin to standard visual working memory studies with simple stimuli (e.g., Luck & Vogel, 1997). As the possibly independent features of real-world objects, we rely on the previous long-term memory work showing that the visual or semantic features people use to recognize which state or pose an object is in (e.g., was the cup full or empty; was the cabinet open or closed) can be forgotten or represented independent of the features people use to distinguish which exemplar of a category they have seen (which cabinet did I see; which mug did I see), as in Utochkin and Brady (2020). Note that although we refer to state and exemplar as object properties, they are not like the simple features of color and orientation often used in working memory tasks. Instead, as noted elsewhere in this article, the visual features used to discriminate them are likely quite complex and different kinds of state changes (i.e., different ways the pose or configuration of an object could be changed) may rely on different visual or semantic features. However, distinguishing between two different states or poses of an object, and between two different exemplars of the same object category are both common and important tasks that need to be regularly performed with real-world objects. Thus, in the present work we focus on these dimensions and ask whether these two aspects of objects are stored in a unitized format in visual working memory. Thus, in all experiments, our participants performed different modifications of the exemplar–state task. This task required participants to report the state (open/closed, full/empty, etc.) of each of two exemplars from the same category that were presented in the working memory display. The idea of this double recognition test was to dissociate exemplar memory and state memory alone from memory for exemplar–state conjunctions (e.g., binding). In Experiments 1 and 2, we asked whether observers are more accurate with exemplar and state memory alone than their conjunction. In Experiment 3, we tested how this relates to spatial location, that is, whether observers might separately update the location of two features when the object moves spatial locations (with only a single feature staying attached to an old location).

## Experiment 1

In Experiment 1, we asked participants to remember four objects in visual working memory: two exemplars from two object categories. The two exemplars from each category could be shown in either the same state or different states. At test, both items of a single category were probed and observers had to recognize the states in which each of the exemplars of that category had been presented. If the information used to discriminate exemplars and the information used to discriminate states are represented in memory in a fully unitized, all-or-none format, we should not observe any differences between the performance of remembering objects in same or different states, because each object would be self-contained, with the features perfectly bound. However, if representations of real-world objects are not unitized and holistic, we anticipate that swap errors could occur between the features of objects (e.g., a mug that was full gets reported as empty because the other mug was empty). Thus, if there is independence in the representation of the features of the objects we predict worse performance for objects presented in different states compared with objects where the two items from the same category are in the same state.

The experiment included two tasks. In the exemplar–state task, we evaluated both whether participants knew which exemplar was in which state (state–exemplar conjunctions), and also whether participants knew whether the two objects had been in the same state or different states than each other (an index of state memory independent of binding). We also measured memory for exemplars alone in a separate exemplar task. Here, participants had to remember two exemplars from two categories (four items in total), but rather than them differing in states and participants needing to recognize the states, instead the test pitted these two previously seen exemplars against two new exemplars from the same category. This task helps to dissociate poor memory for exemplars alone from genuine swap errors.

### Method

#### Participants

Twenty psychology students from the Higher School of Economics, 19 female, age, 18-22 years, *M* = 18.8, took part in the experiment for course credit. All participants reported having normal color vision, normal or corrected-to-normal visual acuity, and no neurological problems. Before the beginning of the experiment, they signed an informed consent form. The sample size was estimated using G*Power 3.1.9.2 (Faul, Erdfelder, Lang, & Buchner, 2009). Our sample size was based on previously reported samples in a similar study of exemplar–state memory (Brady et al., 2013; Utochkin & Brady, 2020)–—15 to 20 participants in one group. The planned sample size also included a few extra participants taking into account the possibility of technical problems or poor performance in some participants. With this sample size, we are able to detect η^2^ equal to 0.08 (for repeated measures analysis of variance [ANOVA]) and Cohen's *d's* equal to 0.7 (two-tailed *t* test) with an α of 0.05 and power (1–β) of 0.8. This is smaller than the effect size reported by previous studies investigating binding errors in visual working memory and visual long-term memory (Bays et al., 2009; Emrich & Ferber, 2012; Pertzov et al., 2012; Utochkin & Brady, 2020), which ranges from 1.1 to 1.9 (Cohen's *dz*).

#### Apparatus and stimuli

The experiment was developed and presented via PsychoPy (Peirce et al., 2019) for Linux Ubuntu. Stimuli were presented on a standard CRT monitor with a refresh frequency of 75 Hz and 1,024 × 768-pixel spatial resolution. Stimuli were presented on a homogeneous white field. Participants sat approximately 47 cm from the monitor. From that distance, the screen subtended approximately 42.4° × 32.5° of visual angle.

Three image sets were used in the experiment. For the exemplar task, we used the image set from the study by Konkle, Brady, Alvarez, and Oliva (2010) including more than 360 categories with 2 to 16 exemplars per category. We selected 120 object categories for the exemplar task. For the exemplar–state task, as the items that tested, we used the image set from the study by Brady et al. (2013; also used by Utochkin & Brady, 2020). It contained 120 unique object categories and each category contained two exemplars (e.g., two different books, Figure 1) and each exemplar was represented by two different states (e.g., open books and closed books, Figure 1). For the items that served as distractors on each trial (e.g.., that were not tested), we created a new image set consisting of 60 categories not overlapping with the categories from Brady et al. (2013) and the categories used in the exemplar task. This image set had the same exemplar–state organization as that of Brady et al. (2013), yet not all categories always had the full set of exemplar and state instances. It was sufficient to have at least one exemplar in one state and another exemplar in a different state, so they could be used as learned but not tested items in different states shown together with two subsequently tested items. For studied but not tested items shown in the same states, we used 60 additional categories from Konkle et al. (2010) not overlapping with those 120 used for the exemplar task (two exemplars were drawn from each category).

##### Sample and test displays

**Figure 2.**
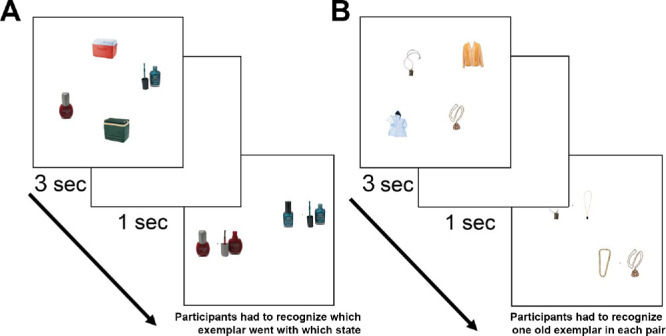
The time course of typical trial in Experiment 1 in (A) the exemplar–state task and (B) the exemplar task. In both tasks, participants had to remember all four initial objects as accurately as possible. Then, after a delay, they had to perform two simultaneous two-alternative forced choice memory tests, picking the correct item at each spatial location. In the exemplar–state task, this meant picking the correct state for each exemplar. In the exemplar task, this meant picking the correct exemplar in each spatial location.

Each to be remembered set (sample) contained four items, each presented at approximately 6.22° × 6.22° of visual angle. The centers of the images laid on an invisible circle with radius 10.3°. The only parameter defining the position of each object was the rotational angle on the imaginary circle. These angles were chosen randomly for each object in each trial with the only restriction that the minimum distance between the centers of any two objects was 30° of rotation. This was done to avoid overlap or clustering between the objects. Two items in the sample were always drawn from one object category and the other two objects were drawn from a different category (Figure 2). At test, two locations corresponding with the centers of two originally presented items—always of the same category—were marked by dots. At 3.3° to the right and to the left of each dot, two images were presented. One of the images at each dot was the exactly the item presented at that location in a sample and another item was always a foil item not presented in the sample. Therefore, one and only one correct answer was present at each probed location and participants had to make two simultaneous two-alternative forced choice judgments. By presenting both tests simultaneously, we decrease the possibility that swap errors arise simply because participants retrieve the wrong exemplar, by making it completely explicit that there are two different relevant items that must be independently remembered.

#### Procedure

The experiment contained two tasks, the exemplar–state and exemplar tasks. The order of the tasks was counterbalanced across participants. During testing, participants were instructed to repeat a syllable “ba” aloud at a rate of about 3 Hz to diminish verbal encoding of stimuli. An experimenter was present in the testing room during the entire experiment and monitored whether the participants followed the instruction to pronounce the syllable.

##### Exemplar–state task

In each trial, two exemplars from two object categories were presented for 3 seconds (e.g., nail polish A opened, nail polish B closed, cooler A closed, and cooler B closed, Figure 2A). This sample display was followed by a 1-second retention interval (blank screen) and then the test screen was presented. Each of the two probed locations contained two possible states of the same exemplar, exactly the exemplar originally presented at that location in the sample (nail polish A opened vs. nail polish A closed at one location; nail polish B opened vs. nail polish B closed at another location, Figure 2A). Participants had to choose the correct state for each exemplar (double two-alternative forced choice). Exemplars of both the subsequently tested and subsequently not-tested category could be presented in either the same states or in different states. There were 60 trials with exemplars from a tested category presented in the same states and 60 with exemplars from the tested category in different states. Whether the nontested exemplars were presented in the same or different states was manipulated orthogonally to the state manipulation of the tested items. Because the participants did not know in advance which exemplars would be tested and because any categorical pair of objects had an equal chance to appear in the same or different states, our participants needed to remember all four items. For each tested category, exemplars were presented in the same states to one-half of the participants and in different states to another one-half of the participants.

##### Exemplar task

The procedure of the exemplar task was similar to that of the exemplar–state task in terms of display structure and time course. The main difference was that two items from the same category were always presented in the same state, and the foil items at test were new exemplars from the same category rather than different states of old exemplars. Therefore, on each trial, observers had to pick a single exemplar at each probed location (Figure 2B). There were 60 trials in the exemplar task.

#### Data analysis

We estimated the overall accuracy (the total number of correctly chosen items) in both the exemplar–state and the exemplar tasks. Report accuracy in the exemplar task was used as a measure of memory for exemplars. Report accuracy in the two conditions of the exemplar–state task was used to estimate the memory for exemplar–state conjunctions. Finally, to estimate state memory, we compared how often the participants reported both items as being in the same states when the studied items had been presented in the same states compared with the trials when the studied items had been presented in different states. This logic was similar to that used by Utochkin and Brady (2020).

We also estimated how often the reported states matched their exemplars in the exemplar–state task. There were three possible outcomes: both correct, one correct, and none correct. If real-world objects are stored in a fully holistic form, we should not observe any difference between these three outcomes as a function of the condition in the exemplar–state task. However, if the features underlying exemplar and state discrimination are stored in some way that is nonholistic, and thus somewhat independent, we should observe an increase in the number of no correct answers for the different states condition, with a concurrent decrease in the number of both correct answers.

The standard frequentist and Bayesian *t* tests were performed. The Bayesian t-test is a direct way to estimate evidence for *H*_1_ against *H*_0_ (Rouder, Speckman, Sun, Morey, & Iverson, 2009). The Bayes factor (BF10) was calculated using JASP 0.9.0.0 (JASP Team, 2018; Wagenmakers et al., 2017) and interpreted using the standard Jeffreys, 1961. Theory of probability (3rd ed.), Oxford University Press, Oxford. The Cauchy distribution with a width of 0.707 was used as a prior distribution of effect sizes under *H*_0_. A Bonferroni correction was made for multiple comparisons in calculating the statistical significance level.

### Results

#### Overall accuracy

**Figure 3.**
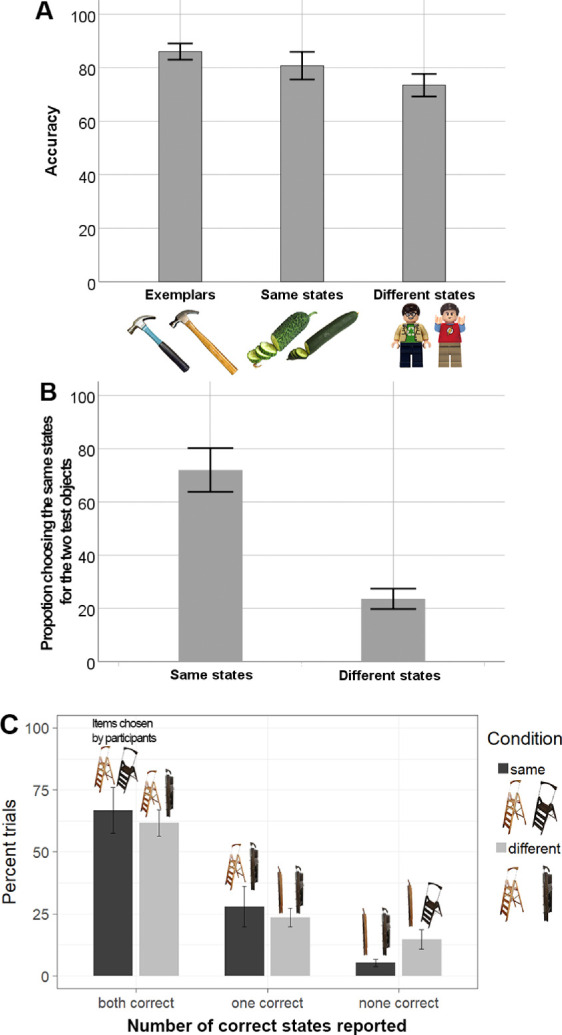
Results for Experiment 1 for overall accuracy (A), state memory accuracy (B) and choosing both, one, or no correct states for exemplars (C). Images on top of the bars in (C) are examples of participants' answers in each of the response outcomes. Note that the axis labels show two the different exemplars of the category (e.g., two different Lego people), either in the same state as each other, or different states than each other. Error bars depict 95% CIs. *LEGO Group. This is an independent site not authorized or sponsored by the LEGO Group*.

A one-way repeated-measure ANOVA was run to compare the total accuracy between the exemplar task and the two conditions of the exemplar–state tasks (same states vs. different states). We found a substantial effect, *F* (2,18) = 28.99, *p* < 0.001, *BF*_10_ > 10^5^, η^2^ = 0.604, Figure 3A. Comparisons between the exemplar task and two conditions of the exemplar–state tasks found differences between all three. The accuracy of exemplar recognition, *M* = 0.86, was higher than the accuracy of state recognition when the objects were presented in same states, *t*(19) = 2.924, *p* = 0.009, Bonferroni corrected α = 0.017, *BF*_10_ = 5.749, Cohen's d = 0.654, and when they were presented in different states, *t*(19) = 7.702, *p* < 0.001, Bonferroni corrected α = 0.017, *BF*_10_ > 10^5^, Cohen's d = 1.722. Most important, the accuracy of reporting correct exemplar–state conjunctions, *M* = 0.81, was greater in trials when two exemplars were presented in the same states compared with trials when they were presented in two different states, *M* = 0.74; *t* (19) = 4.772, *p* < 0.001, Bonferroni corrected α = 0.017, *BF*_10_ = 216, Cohen's d = 1.067, Figure 3A.

#### State memory

The percentage of time participants choose two responses in the same states is our index of state memory. We found that participants did so more often when the objects had actually been presented in the same states compared with when they had been presented in different states, same states: *M* = 0.72, different states: *M* = 0.23; comparison: *t* (19) = 10.7, *p* < 0.001, *BF*_10_ > 10^6^, Cohen's d = 2.393. This finding suggests that our participants had good memory for which states were presented, regardless of their ability to report which exemplars these states belonged to.

#### Accuracy of conjunction memory within paired choices

Given the good memory for whether two exemplars had been presented in the same or in different states, we can ask how often these memories were correctly bound to the exemplars. To estimate that, we analyzed the proportions of three possible outcomes of the paired choices that our observers made on each trial: both states correct, one state correct, or zero correct. Swap errors occur if participants often report both items incorrectly (e.g., knows the items had different states but not which state went with which exemplar). Thus, an excess of 0 correct trials is evidence of swap errors (e.g., knowledge of the states separate from the exemplars they go with).

If the overall accuracy is simply impaired by the objects being in different states (perhaps from greater difficulty encoding them, for example), with no change in binding difficulty per se, participants overall performance should predict their performance for picking both items wrong. For example, with an 81% overall correct response rate, as in the same state condition, if the two objects are responded to independently we expect the proportion of trials with two correct answers to be (0.81)^2^ (e.g., an independent chance to get each object correct); with item one correct and item two incorrect to be (0.81)(1 – 0.81); with item one incorrect and item two correct to be (1 – 0.81)(0.81); and with zero correct answers to be (1 – 0.81)^2^. The small decrease in percent correct to 74% in the different state condition can also be analyzed in this way, to see if relatively speaking, participants make more double errors (swaps) than would be expected given how often they get none and one correct. These errors should change predictably if the only difference in conditions is an overall accuracy effect from more difficult encoding rather than independent knowledge of states and exemplars (resulting in a change from [1 – 0.81]^2^ = 3.6% zero correct trials, to [1 – 0.74]^2^ = 6.7% zero correct trials). By contrast, if different states result in binding difficulties, then when the objects are presented in different states, participants should show an abundance of zero correct trials, which represent swap errors (where they knew the states, but not how they went together).

Overall, we found that there was no significant difference between the proportions of choosing both correct answers between the two conditions of exemplar–state task, same states: *M* = 0.67, different states: *M* = 0.62; comparison: *t*(19) = 1.609, *p* = 0.124, *BF*_10_ = 0.698, Cohen's d =.360, and also between the proportions of choosing only one correct answer, same states: *M* = 0.28, different states: *M* = 0.24, comparison: *t*(19) = 1.073, *p* = 0.297, *BF*_10_ = 0.386, Cohen's d = 0.240, because both displayed small decreases that did not reach significance. However, there were significantly more none correct trials for objects shown in different states compared with same states, same states: *M* = 0.05, different states: *M* = 0.15; comparison: *t* (19) = 5.345, *p* < 0.001, *BF*_10_ = 676.7, Cohen's d =1.195. This result is indicative of swap errors.

We can also compare this level of no correct answers with the independent responses baseline. In the same state condition, there was no significant excess of zero correct answers relative to the individual performance level, same states none correct vs. predicted from individual performance level: *t*(19)=0.468, *p* = 0.645, *BF*_10_ = 0.256, Cohen's d = 0.11. However, for the different state condition there was a significant excess of such trials, different states none correct vs. predicted from individual performance level: *t* (19) = 7.14, *p* < 0.001, *BF*_10_ > 10^5^, Cohen's d = 1.59, consistent with swaps. This is because zero correct choices in the different state condition mean that a participant reported the states as being different (which is correct in terms of states alone) but ascribed them to the wrong exemplars (which is incorrect in terms of conjunctions or swap errors). In comparison with the failure to report any correct conjunctions for same state objects (which is more consistent with the absence of state memory), the “swap responses, according to our analysis, are observed in a considerable number of trials.

Overall, our results showed that our observers were quite good at recognizing exemplars and at discriminating whether the objects were presented in the same or different states. However, in a significant number of trials, they showed difficulties with reporting correct exemplar–state conjunctions. As a specific sign of a binding failure, this difficulty manifested as an increased fraction of trials within the different state condition where observers successfully reported the states as being different but chose wrong the exemplars for these two states. Note that the failure to report any conjunction correctly is rare in the same state trials where people do not actually need to remember exact conjunctions to perform the exemplar–state task and an ability to report the conjunction depends on memory only for the state itself. Therefore, we conclude that the difference between memory performance in the two conditions of the exemplar–state task is a result of binding failures, which is consistent with nonholistic, at least partially independent storage of exemplar and state features of real-world objects.

## Experiment 2

The exemplar and the exemplar–state tasks were separated into two different blocks in Experiment 1. This practice could artificially encourage our observers to particularly focus on exemplar or state features during encoding, which could result in an inflated rate of swap responses and overestimate the independence of exemplar and state memories. Therefore, in Experiment 2, we randomly mixed trials from the exemplar and exemplar–state tasks to discourage our participants from selective encoding of the corresponding features.

### Method

#### Participants

Twenty-five psychology students from the Higher School of Economics, 21 female; age, 18 to 33 years; *M* = 19.7, took part in the experiment for course credit. All participants reported having normal color vision, normal or corrected-to-normal visual acuity, and no neurological problems. The apparatus, stimuli, and procedure were the same as in Experiment 1. The main difference from Experiment 1 is that trials from the exemplar task were randomly mixed with trials from exemplar–state task.

### Results

One participant showed less than 50% accuracy in all conditions and was excluded from the analysis.

#### Overall accuracy

**Figure 4.**
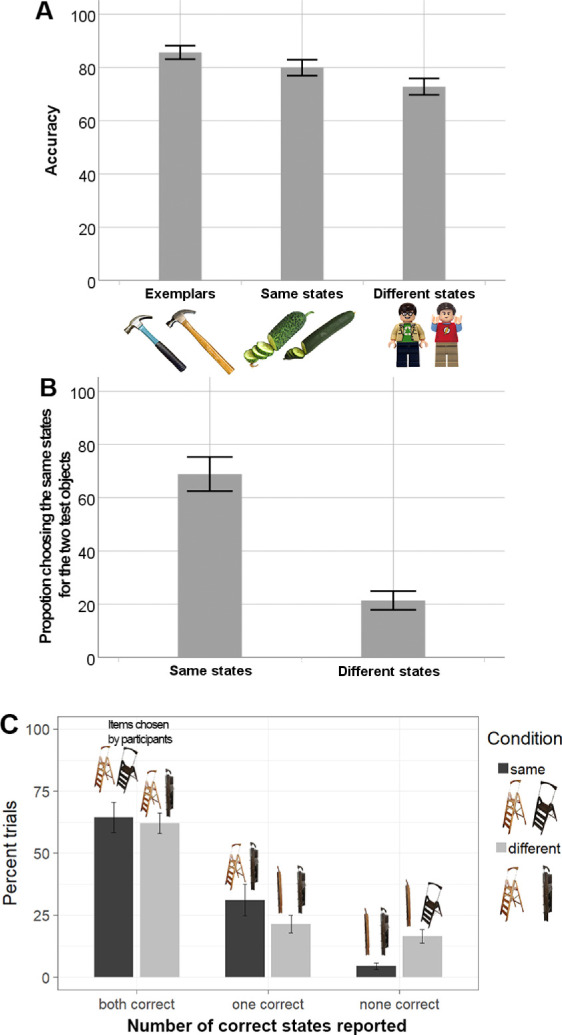
Results for Experiment 2 for overall accuracy (A), state memory accuracy (B), and choosing both, one, or no correct states for exemplars (C). Images on top of the bars in (C) are examples of participants' answers in each of the response outcomes. Note that the axis labels show two the different exemplars of the category (e.g., two different Lego people), either in the same state as each other, or different states than each other. Error bars depict 95% CIs. *LEGO Group. This is an independent site not authorized or sponsored by the LEGO Group*.

We found evidence for a strong effect of the task and same/different-state manipulation on recognition accuracy, *F* (2,21) = 39.23, *p* < 0.001, *BF*_10_ > 10^8^, η^2^ = 0.630 (Figure 4). Participants were more accurate in the exemplar task, *M* = 0.86, than in the exemplar–state task, both when the states were same, *M* = 0.80; comparison: *t*(23) = 4.247, *p* < 0.001, Bonferroni corrected α = 0.017, *BF*_10_ = 100.2, Cohen's d = 0.867, and when the states were different, *M* = 0.73; comparison: *t*(23) = 9.024, *p* < 0.001, Bonferroni corrected α = 0.017, *BF*_10_ > 10^6^, Cohen's d = 1.842. Most important, within the exemplar–state task, our participants were more accurate when tested exemplars were shown in the same state, *M* = 0.80, than when they were in different states, *M* = 0.73; comparison: *t*(23) = 4.512, *p* < 0.001, Bonferroni corrected α = 0.017, *BF*_10_ = 180.5, Cohen's d = 0.921.

#### State memory

Like in Experiment 1, our participants were quite good at discriminating whether the two tested exemplars had been presented in the same or different states. For objects presented in the same states, they chose two response options of the same states much more frequently, *M* = 0.69, than when the objects had been presented in different states, *M* = 0.21; comparison: *t*(23) = 13.93, *p* < 0.001, *BF*_10_ > 10^8^, Cohen's d = 2.843.

#### Accuracy of conjunction memory within paired choices

Our participants chose both correct exemplar–state conjunctions with approximately equal frequencies in the two conditions of the exemplar–state task, same states: *M* = 0.64, different states: *M* = 0.62; comparison: *t*(23) = 0.747, *p* = 0.463, *BF*_10_ = 0.276, Cohen's d =.152. The participants chose only one correct conjunction for items shown in same states more frequently than for items shown in different states, same states: *M* = 0.31, different states: *M* = 0.21, comparison: *t*(23) = 2.649, *p* = 0.014, *BF*_10_ = 3.581, Cohen's d = 0.541. Critically, they were substantially more likely to choose no correct conjunctions for items shown in different states than for items shown in same states, same states: *M* = 0.05, different states: *M* = 0.17, comparison: *t* (23) = 7.846, *p* < 0.001, *BF*_10_ > 10^6^, Cohen's d = 1.6. Again following the logic of what is expected from independent responses based on participants’ individual percent correct, we find an excess of none correct trials in the different state condition only, comparison for same states: *t*(23) = 0.008, *p* = 0.994, *BF*_10_ = 0.215, Cohen's d = 0.002; comparison for different states: *t*(23) = 10.676, *p* < 0.001, *BF*_10_ > 10^7^, Cohen's d = 2.18. This result replicates the finding from Experiment 1, showing that, in a substantial number of trials with a correct report about tested items being in different states, participants committed swap errors failing to correctly report which exemplars these states had gone with, but being—correctly—aware that there were two different states present.

The results of Experiment 2 closely replicated the pattern of results from Experiment 1. In both experiments, there were swap errors, where participants failed at reporting the state–exemplar conjunctions but had accurate memory for whether the objects’ states had been the same or different. Because the mixed design of Experiment 2 discouraged selective encoding of exemplar features or state features, it seems that swap errors are not strongly dependent on an encoding strategy. These results are compatible with the idea of there being significant independence in the features that underly exemplar and state discrimination in visual working memory.

#### Robustness across categories in Experiments 1 and 2

To test whether the differences between the two conditions of the exemplar–state task are caused by our central manipulation of same versus different state, not by internal characteristics of individual images potentially affecting the memorability of objects, we analyzed the proportions of correct answers and the likelihood of choosing the same states across all tested images. In other words, we treated categories as a random effect rather than participants to ensure robustness not only across participants, but also across individual images. In particular, we can estimate how many observers chose the same states and correct exemplar–state conjunctions for every given category as a function of whether items in this category were presented in the same or different states (rather than how many observers did so, regardless of category). As the results of Experiments 1 and 2 showed highly similar patterns, we merged responses from all observers taking part in these two experiments. Overall, each category was seen by 22 participants in the same state and by 22 participants in different states.

We found that the overall accuracy across categories was lower when objects from these categories were shown in different states than when they were shown in same states, *t*(119) = 6.180, *p* < 0.001, Cohen's d = 0.564, *BF*_10_ > 10^6^. The probability of choosing the same states for objects shown in different states was lower than the probability of choosing same states for objects shown in same states, *t*(119) = 26.52, *p* < 0.001, Cohen's d = 2.421, *BF*_10_ > 10^40^. Therefore, our pattern of results shows the robustness of the pattern described in Experiments 1 and 2 not only across observers but also across stimuli.

#### The effect of not tested states in Experiments 1 and 2

In each trial of our exemplar–state task, we tested only one category (e.g., two of the four objects), and the main manipulation was whether these objects were in the same state as each other or different states. However, items that have not been tested could also appear in either the same or different states. Because the same versus different status of the states of the not tested items were purposefully made orthogonal to the states of the tested items, it is possible to estimate the contribution of the former to the accuracy of reporting the latter. Given that performance estimates for such an analysis were built on 30 trials per combination of tested and not tested states (rather than 60 trials for our main analysis), we merged the data from Experiments 1 and 2 to compensate for some possible loss in the precision of individual estimates owing to the reduced number of trials.

##### Overall accuracy

**Figure 5.**
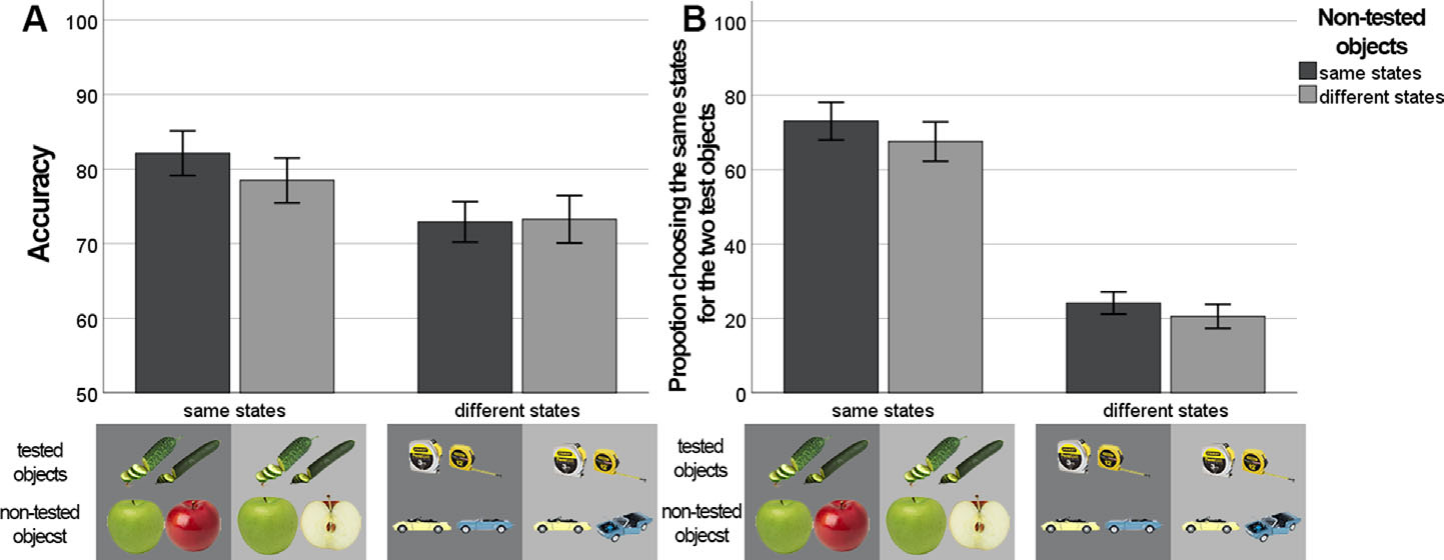
Results for the influence of states of tested and not tested objects on overall accuracy (A), state memory accuracy (B). Error bars depict 95% CIs.

A repeated-measure two-way ANOVA was run to estimate the effect of state manipulations in tested and not-tested items. We found a significant effect of the states of tested items on accuracy, *F*(1,43) = 43.305, *p* < 0.001, *BF*_10_ > 10^7^, η^2^ = 0.502 (Figure 5), reflecting the trend reported separately for each experiment: observers were less accurate when tested items were presented in different states. More importantly, we found evidence for the effect of tested items states × not tested items states on accuracy, *F*(1,43) = 5.429, *p* = 0.025, *BF*_10_ =1.02, η^2^ = 0.112. This effect arose because the accuracy of reporting tested conjunctions presented in same states was lower when the not-tested objects were presented in different states compared with not-tested objects presented in same states, *t*(43) = 2.991, *p* = 0.005, Cohen's d =.451, *BF*_10_ = 7.731 (Figure 5A). When the tested objects were presented in different states, the states of not tested objects had no effect, *t*(43) = 0.206, *p* = 0.838, Cohen's d =.031, *BF*_10_ = 0.167. In other words, there was an overall performance improvement particularly in the condition where both categories were more homogenous, each having only one state present. This finding is broadly consistent with the idea that state and exemplar properties are not automatically represented in a single holistic representation, but that binding the features used to discriminate state and exemplar features is difficult.

##### State memory

Participants reported the tested items were in the same states less when the tested objects were in different states compared with same states, *F* (1,43) = 305.784, *p* < 0.001, *BF*_10_ > 10^54^, η^2^ = 0.877, reflecting the result shown separately in Experiments 1 and 2. Importantly, the percentage reporting both tested items were in the same state was lower when not-tested objects were in different states compared with when the not tested objects were in the same states, *F* (1,43) = 22.424, *p* < 0.001, *BF*_10_ = 0.282, η^2^ = 0.343 (Figure 5B). Therefore, we found that the states of not-tested but also memorized objects had a slight effect on reported states of tested objects in the same direction as the effect of tested objects states. Again, the finding that observers’ answers were sensitive to state manipulations (although less than within the tested categories) supports the idea of somewhat independent storage of the features underlying state and exemplar discrimination in real-world objects.

#### Similarity between exemplar and state pairs

**Figure 6.**
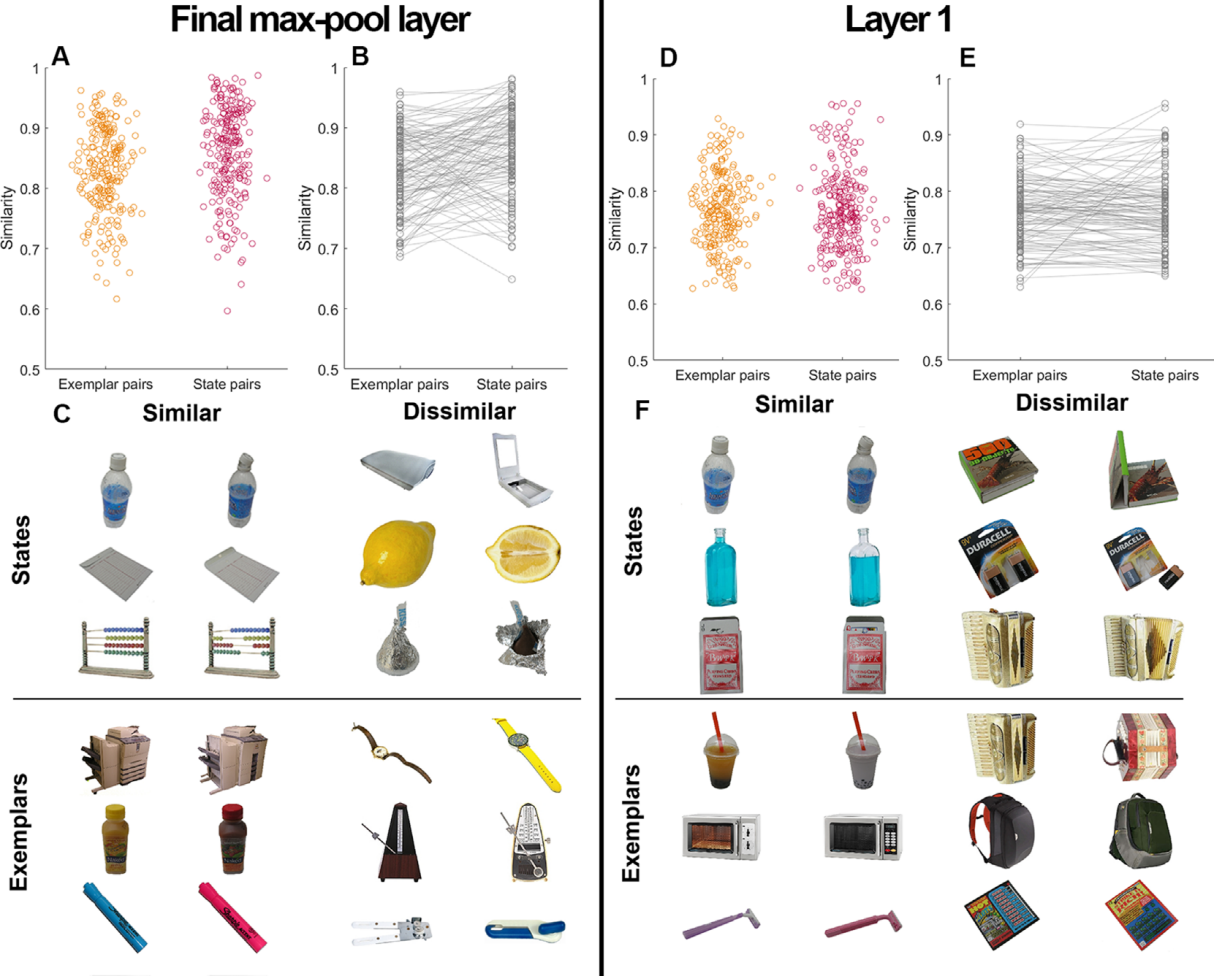
Similarity of exemplar and state pairs (0 = completely dissimilar objects; 1 = completely similar objects). Final max-pool layer: Estimation of similarity for paired exemplars and paired states (A) and average values for each category show significant heterogeneity by category (B). The examples of most similar and dissimilar pairs of exemplars and states according to the max pool layer. (C). Layer 1: Estimation of similarity for paired exemplars and paired states (D) and average values for each category show significant heterogeneity by category (E). The examples of most similar and dissimilar pairs of exemplars and states according to layer 1 (F).

Looking at the performance on the exemplar task compared with the exemplar–state task, one could argue that there are differences in the exemplar and state manipulations themselves that account for this effect. For example, it intuitively seems that two different states of the same object might be more visually similar than two different exemplars in the same state, and that this could affect the two-alternative forced choice task performance (e.g., Brady & Störmer, 2020). In this case, the exemplar–state task would be harder than the exemplar task based on the images alone, rather than because of binding difficulties. Although previous work has found these two kinds of test tend to be similar in difficulty (Brady et al., 2008), to test this intuition more systematically, we quantitatively estimated similarity using the VGG16 pretrained convolutional neural network (Brady & Störmer, 2020; Simonyan & Zisserman, 2014). We were particularly focused on the top max-pool layer, which allowed us to retrieve high-level features that are more invariant to low-level transforms, because previous work demonstrates that this provides a useful proxy for object similarity (Brady & Störmer, 2020) with extremely similar object stimuli. However, we also estimated similarity values based only on layer 1, which are more related to very low-level image similarity and should have little invariance. We used our target stimuli (Brady et al., 2013) and estimated similarity between paired exemplars in the same states (exemplar pairs) and between the two states of the same exemplar (state pairs). Similarity for the 240 exemplar pairs and 240 state pairs (Figure 6A) and the average similarity for each category (Figure 6B) were estimated.

We found quite small (approximately 3%) but significant differences in the similarity based on final max-pool layer estimates between exemplar pairs, *M* = 0.83, and state pairs, *M* = 0.86; comparison: *t* (119) = 4.272, *p* < 0.001, *BF*_10_ = 411.6, Cohen's *d* = 0.39 (Figure 6B), with state pairs being slightly more similar on average. However, we found no significant differences in the similarity based on layer 1, exemplar pairs: *M* = 0.77; state pairs: *M* = 0.77; comparison: *t* (119) = 0.314, *p* = 0.754, BF_10_ = 0.1, Cohen's *d* = 0.029) (Figure 6E). We also found significant, although weak, correlations between the similarity estimates based on the final max-pool layer and the similarity estimates based on layer 1, exemplar pairs: *r* (118) = 0.39 *p* < 0.001; state pairs: *r* (118) = 0.44 *p* < 0.001. To estimate the effect of similarity on task performance—and validate the similarity metric—we calculated a correlation between the average performance on the task and the similarity estimates for exemplars and state pairs based on both final max-pool layer and layer 1. We found that layer 1 did not significant predict performance in the task (*p* > 0.10), but that the max pool layer did, with a significant negative correlation between similarity values for state pairs based on the final max-pool layer and accuracy, *r* (118) = 0.31 *p* < 0.001. Therefore, state similarity—as measured by the more invariant max pool layer—indeed affected overall performance and the difficulty of the two-alternative forced choice task (e.g., Brady & Störmer, 2020).

Thus, to test whether these differences in similarity affected the comparisons we made between the exemplar task and the exemplar–state task, we selected the categories with most similar exemplars and most dissimilar states, according to the max pool layer (we chose categories where exemplar similarity was higher than state similarity, 40 categories overall with exemplar similarity of 0.87 and state similarity of 0.81) and redid the analysis from Experiments 1 and 2. In this sample, the states should actually be “easier” to discriminate than the exemplars. Thus, this provides a strong test of whether similarity alone accounts for the difference between our conditions.

##### Overall accuracy

Observers were less accurate when exemplars were in different states, *M* = 0.75, compared with same states, *M* = 0.84; comparison: *t*(43) = 4.305, *p* < 0.001, Cohen's *d* = 0.649, *BF*_10_ = 249.

##### State memory accuracy

Observers reported that exemplars were in the same states more frequently when exemplars were presented in the same states, *M* = 0.75, compared with different states, *M* = 0.20; comparison: *t*(43) = 17.352, *p* < 0.001, Cohen's *d* = 2.616, BF_10_ > 1017.

##### Accuracy of conjunction memory within paired choices

There were no differences between conditions in choosing both correct, same states: *M* = 0.72, different states: *M* = 0.66, comparison: *t*(43) = 1.985, *p* = 0.054, Cohen's *d* = 0.299, BF_10_ = 0.975, and one correct, same states: *M* = 0.25, different states: *M* = 0.19, comparison: *t*(43) = 1.811, *p* = 0.077 Cohen's *d* = 0.273, BF_10_ = 0.729. The percentage of none correct answers was significantly higher in the different states condition compared with the same states condition, same states: *M* = 0.04, different states: *M* = 0.15, comparison: *t*(43) = 6.046, *p* < 0.001, Cohen's *d* =.911, BF_10_ = 49,614.

This additional analysis demonstrates the same pattern as our main analysis, even for objects with highly dissimilar states. This finding suggests that binding errors still occur when discrimination at test is made easier. Most important, the patterns are preserved even when the state pairs are more dissimilar than the exemplar pairs. Therefore, inferior performance in the exemplar–state task cannot be explained by interobject similarity. Instead, we conclude that it has to do with the need to report exemplar–state conjunctions.

## Discussion of Experiments 1 and 2

In Experiments 1 and 2 we found that our participants were less accurate at reporting the conjunctions of exemplars and states when these exemplars had been shown in different states compared with exemplars shown in the same state. This decrement in conjunction recognition was combined with good recognition memory for exemplars and good ability to discriminate—without the need for binding—whether states were the same or different. Importantly, the accuracy decrement that we observed for objects presented in the different states was mostly provided by trials where observers correctly reported the states being different, but swapped these states between exemplars. Moreover, we found some benefits for performance when the nontested objects were in the same states rather than different states, suggesting the task was easier when the nontested objects did not require binding states to exemplars. Therefore, we conclude that the features underlying exemplar and state discrimination are represented in some sense independently and that remembering their conjunctions causes additional difficulty compared with remembering these features per se. A fully unitized, all-or-none, totally bound representation account fails to account for our results, because it predicts that the working memory traces should be indifferent to whether two different exemplars are presented in same or different states: in any case, two separate records are created with equal likelihood to be stored or forgotten.

In fact, binding was also not necessary in the same states condition to recall both exemplar–state conjunctions. It was sufficient to remember a common state for a category instead, which is also consistent with the idea of independence, rather than each object being a holistic, unitized object encoded separately from each other. In Experiment 1, observers could discard exemplar information in the state–exemplar condition and remember only the common state, as the exemplar memory was not tested in the same block as state memory. However, in Experiment 2 with its mixed design observers did not know in advance whether their exemplar or exemplar–state memory would be tested. The nearly identical results of Experiments 1 and 2 suggests, therefore, that observers were encoding relevant details sufficient to discriminate both state and exemplar comparisons in both experiments. However, encoding the relevant features for both (I remember both these mugs and that they both were full) does not seem to entail that these features were fully unitized and bound (if I remember these mugs, I also remember that they were full, and vice versa). The results from the different state condition demonstrate that while participants appear to encode all the relevant features in both Experiments 1 and 2, these features are nevertheless somewhat independent (e.g., I sometimes remember the mugs and I remember one full and one empty, but I do not remember which one was full and which was empty). In the General Discussion, we consider possible exemplar and state representations leading to the observed pattern of results in more detail.

## Experiment 3

In Experiment 3, we tested another interesting prediction following from the idea of at least partially independently stored properties of real-world objects. In particular, we looked at how people update previously studied information, one of the crucial functions of working memory (Ecker, Lewandowsky, Oberauer, & Chee, 2010; Nyberg & Eriksson, 2016). If a task requires participants to remember an item and then update it, taking into account a subsequent change to the item (update) can often cause confusions between the initial and updated representations, resembling binding errors (Gorgoraptis, Catalao, Bays, & Husain, 2011). An example of such errors in visual working memory can be a failure to completely update location changes during retention. Hollingworth and Rasmussen (2010) showed that binding to new locations after motion is nevertheless impacted by a remaining binding to the original locations. Other work on the spatial congruency bias also suggests that a location of an object is automatically attended and that the identity of an object is bound to this location even after updates (Bapat, Shafer-Skelton, Kupitz, & Golomb, 2017; Golomb, Kupitz, & Thiemann, 2014; Shafer-Skelton, Kupitz, & Golomb, 2017). With respect to our main research interest, this point raises an important question about real-world object representation: When an object changes location, will observers update or fail to update the entire set of object properties to a new location? Or is it possible that separate properties can separately fail to be updated? For example, imagine I am shown a full coffee mug *A* in a location *X* and an empty coffee mug *B* at a location *Y*. If my memory for the mug *A* is then tested at the location *Y* (originally belonging to the mug *B*), will I fail to update both the mug *A* and its “fullness” (as expected if updating is based on unitized memories) or I can update the mug *A* but remember the emptiness encoded from that location (which should cause a swap report as we defined it in Experiment 1)? We addressed this question in Experiment 3. We tested whether observers commit more swaps between exemplars and states of real-world objects when updating of locations is required. In particular, we tested whether observers more often choose the wrong state for a studied exemplar if at test it takes the location of a different exemplar shown in a different state. If features can be independently bound to a certain location, we predict that we will find no difference between original and updated locations for exemplars in the same states, but will find them for exemplars in the different states. This prediction follows the similar logic for Experiments 1 and 2. In other words, if two exemplars in the same state change their locations at test it should cause no confusions, regardless of whether the state is updated or not, because of the commonality between the two states at the two locations. By contrast, if exemplars in different states change their locations at test, this could cause more binding errors if updating is independent for state information (e.g., I remember a full mug at this location but do not remember which mug it was, so I choose a full mug here). By contrast, if updating works on unitized, fully bound representations then we expect that exemplar swap at test should produce an effect on exemplar–state reports both when the states are same and when they are different.

### Method

#### Participants

Twenty-five psychology students from the Higher School of Economics, 22 female; age, 18 to 22 years, *M* = 19.24, took part in the experiment for extra course credits. All participants reported having normal color vision, normal or corrected to normal visual acuity, and no neurological problems.

The apparatus, stimuli, and spatial layout were similar to Experiments 1 and 2. Three image sets were used in this experiment: 120 categories from an image set (Brady et al., 2013) and 40 categories from an image set created by ourselves were used as tested categories in the exemplar–state task. Eighty-two categories from our image set and 78 categories from (Konkle et al., 2010) were used as nontested categories in the exemplar–state task.

#### Procedure

**Figure 7.**
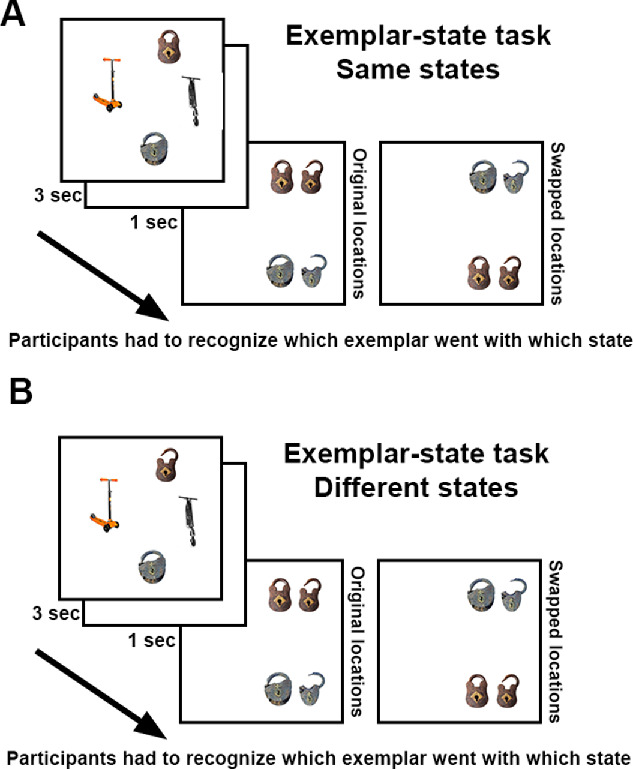
Example trials of Experiment 3 where two exemplars (locks) are (A) studied in the same states and tested in their original locations, or (B) studied in different states and tested in swapped locations.

In this experiment, we used only the exemplar–state task. As in Experiments 1 and 2, observers had to remember two pairs of exemplars from two object categories and each pair of exemplars could be presented in the same or different states (Figure 7). The critical difference from Experiments 1 and 2 was at test. In one-half of trials, the presented exemplars from the tested category were tested at their original locations. If exemplar *A* was shown at location *X* and exemplar *B* was shown at location *Y*, then at test the old and new states of exemplar *A* were also shown at location and the old and new states of exemplar *B* were also shown at location *Y* (Figure 7), as in the previous experiments. In another one-half of the trials, the two tested exemplars swapped their locations. If exemplar *A* was studied at location *X*, its new and old states were tested at location *Y*, and if exemplar *B* was studied at location *Y* its states were tested at location *X* (Figure 7). Participants were warned that objects could swap their locations at test and were instructed to recognize in which state each exemplar in the category was presented, regardless of test location.

In 80 trials, the tested exemplars were studied in different states and in 80 trials the tested exemplars were studied in the same states. In 80 trials, objects were tested in their original locations and in the other 80 trials in swapped locations. Categories were counterbalanced across conditions between participants using a Latin square.

#### Design and analysis

We had a 2 (objects in same and different states) ✕ 2 (original or swapped test locations) within-subject design. We estimated the overall accuracy (total number of correctly chosen items) and state memory for the two location conditions (original vs. swapped) and for same versus different states. We also estimated the accuracy of conjunction memory within paired choices similarly to Experiments 1 and 2. For Bayesian *t* tests the same priors as in Experiments 1 and 2 were used.

### Results and discussion

#### Overall accuracy

**Figure 8.**
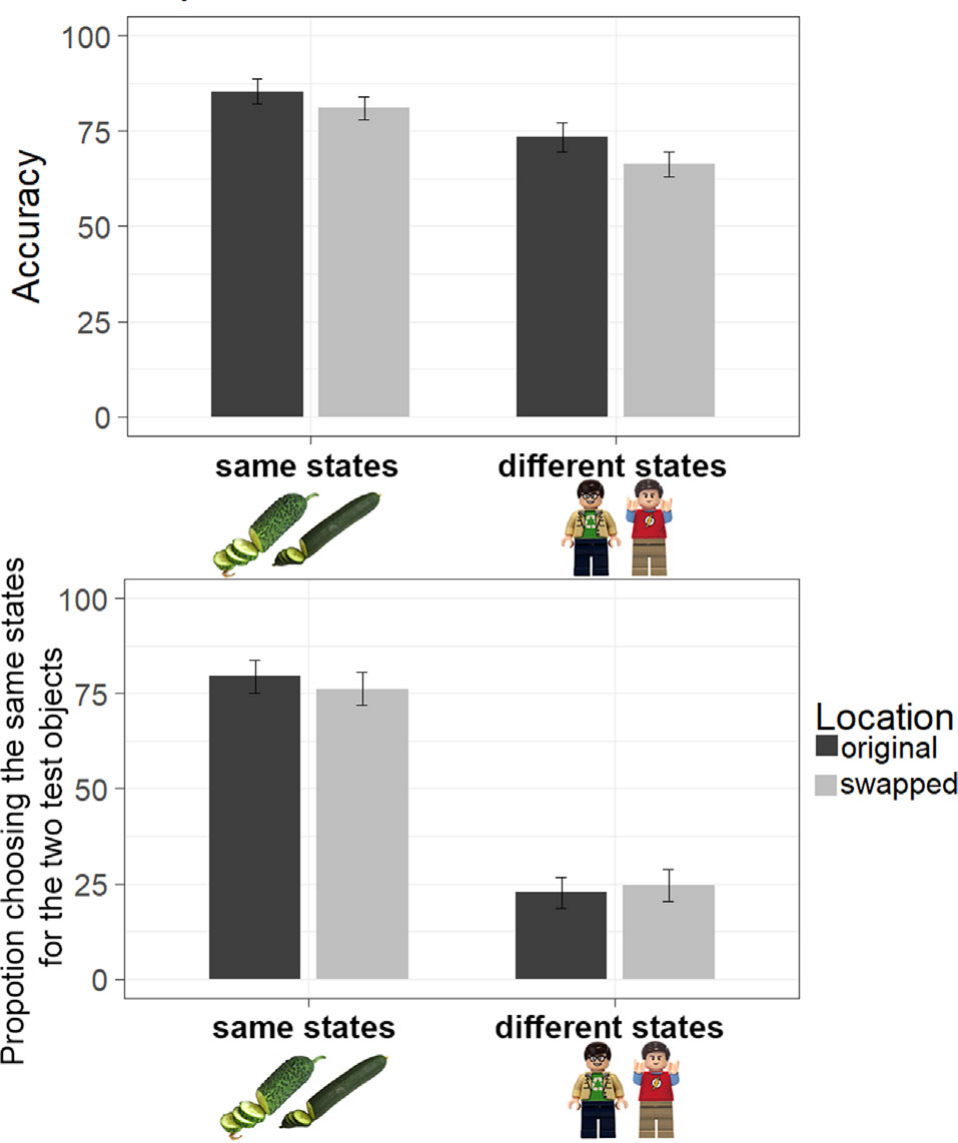
Results for Experiment 3 for overall accuracy and state memory for objects in same and different states. Error bars depict 95% CIs. *LEGO Group. This is an independent site not authorized or sponsored by the LEGO Group.*

A two-way repeated-measures ANOVA was run to estimate the effects of studied exemplars being in the same versus different states and of test location. We found an overall effect of test location, *F* (1,24) = 30.230, *p* < 0.001, *BF*_10_ = 12.678, η^2^ = 0.557. Observers were overall less accurate reporting exemplar–state conjunctions at swapped locations (Figure 8). We also found a strong effect of the studied states, *F* (1,24) = 117.380, *p* < 0.001, *BF*_10_ > 10^10^, η^2^ = 0.830. Observers were less accurate when objects were presented in different states compared with same states (Figure 7), which is in line with the corresponding findings from Experiments 1 and 2. However, we found no evidence for the interaction effect between studied states and test location, *F* (1,24) = 1.896, *p* = 0.181, *BF*_10_ = 0.517, η^2^ = 0.073. That is, participants did not seem to have additional difficulty recognizing the different state items at updated locations compared with the same states at updated locations, broadly consistent with whole-object updating rather than separate updating for each property.

#### State memory

There was a strong effect of the state of studied objects, *F* (1,24) = 288.742, *p* < 0.001, *BF*_10_ > 10^10^, η^2^ = 0.923. Participants more often chose two same states when objects were presented in the same states and this effect was flipped for objects presented in two different states. We found no evidence of the effect of test location, *F* (1,24) = 0.563, *p* = 0.46, *BF*_10_ = 0.208, η^2^ = 0.023, but did find a weak test location ✕ studied states interaction, *F* (1,24) = 6.197, *p* = 0.02, *BF*_10_ = 0.128, η^2^ = 0.205, consistent with less accurate memory for states when the items flipped location (e.g., both conditions being closer to 0.50).

#### Accuracy of conjunction memory within paired choices

**Figure 9.**
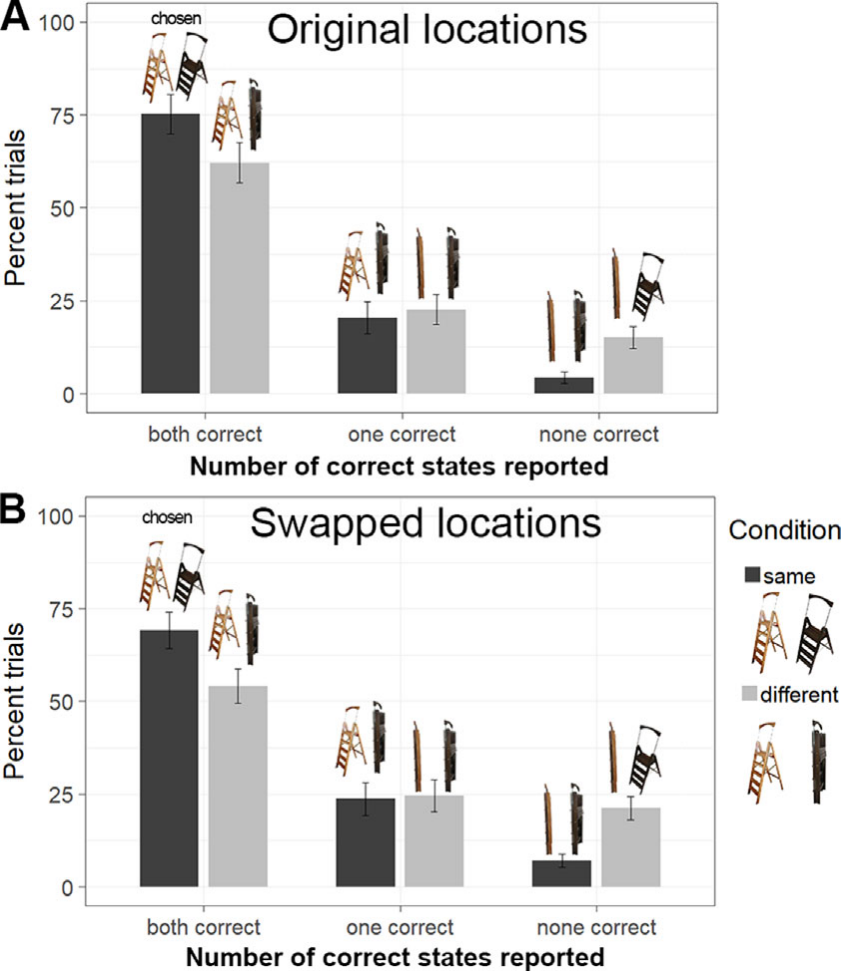
Results for Experiment 3 for choosing both, one or none correct states for exemplars tested in (A) original locations and (B) swapped locations. Error bars depict 95% CIs.

We analyzed the frequencies of paired report outcomes (both, one, or none correct) separately for original (Figure 9A) and swapped locations (Figure 9B). Overall, the patterns were strongly similar across these two conditions. Participants more frequently chose both of the correct exemplar–state conjunctions when the objects has been shown in the same state compared with different states, original locations: *M* = 0.75 for same states, *M* = 0.62 for different states; comparison: *t*(24) = 6.381, *p* < 0.001, *BF*_10_ = 13,605, Cohen's d = 1.276; swapped locations: *M* = 0.69 for same states, *M* = 0.54 for different states; comparison: *t*(24) = 5.918, *p* < 0.001, *BF*_10_ = 4,811, Cohen's d = 1.184. This result was well-mirrored by the pattern of choosing no conjunctions correct: Participants were more likely to choose no correct conjunctions for items shown in different states than for items shown in same states, original locations: *M* = 0.04 for same states, *M* = 0.15 for different states; comparison: *t* (24) = 7.712, *p* < 0.001, *BF*_10_ > 10^6^, Cohen's d = 1.542; swapped locations: *M* = 0.07 for same states, *M* = 0.21 for different states; comparison: *t* (24) = 8.067, *p* < 0.001, *BF*_10_ > 10^6^, Cohen's d = 1.613. There were no differences between the proportions of choosing only one correct conjunction between the two state combinations, original locations: *M* = 0.20 for same states, *M* = 0.23 for different states; comparison: *t*(24) = 0.912, *p* = 0.371, *BF*_10_ = 0.307, Cohen's d =.182; swapped locations: *M* = 0.24 for same states, *M* = 0.25 for different states; comparison: *t*(24) = 0.358, *p* = 0.723, *BF*_10_ = 0.224, Cohen's d = 0.072. This result replicates the corresponding pattern from Experiments 1 and 2 showing that observers tend to swap states between exemplars when these exemplars are presented in different states, with no additional reliable effect of switching location.

Thus, in Experiment 3 we strongly replicated the basic pattern from Experiments 1 and 2 that observers show better memory for states (whether these states are same or different) than for exemplar–state conjunctions (which state goes with which exemplar), especially when conjunction discrimination is critical for doing the task as in the case of exemplars presented in different states. Therefore, Experiment 3 confirms the robustness of this basic pattern indicating the relative independence of internal object features (features underlying exemplar and state discrimination). In addition to this point, we found some cost of location swaps that can be interpreted in terms of updating failures. This result replicates the previously reported tendency of an object to be bound to its original location after motion or during the update (Bapat et al., 2017; Golomb et al., 2014; Hollingworth & Rasmussen, 2010; Shafer-Skelton et al., 2017). The cost of location swaps was not very strong (difference of approximately 6% between original and swapped locations), which is also consistent with the previous demonstrations (e.g., approximately 4% between original and updated locations in Hollingworth & Rasmussen, 2010).

Having succeeded with inducing updating failures, we can turn to the main question of Experiment 3. According to our prediction, independent updating of different features could be inferred only if we found no impairment for objects presented in the same states and found this impairment in the different states condition. Because we found no difference in the amount of updating failures for exemplars shown in same versus different states, we conclude that state features are not bound to locations independently from exemplar features. Instead, it seems that location updating has something to do with the whole object representation. This finding is consistent with the previous demonstrations and the object file theory (Bapat et al., 2017; Golomb et al., 2014; Hollingworth & Rasmussen, 2010; Shafer-Skelton et al., 2017; Kahneman, Treisman, & Gibbs, 1992), and especially consistent with recent work from Dowd and Golomb (2019) showing that updating items does not break binding in the case of simple objects.

Thus, on the one hand, we see that the features underlying state discriminations behave relatively independently from those underlying exemplar discriminations, as revealed by the same versus different states manipulation. On the other hand, location updating appears to act on the entire set of object features.

## General discussion

Experiments 1 and 2 showed that real-world objects are not necessarily stored as completely unitized, fully bound units in visual working memory, as swap errors occur between features of different objects of the same category. These results are in line with theories based on simple features which argue that visual working memory is not based on fully bound representations (e.g., with the weak object hypothesis, Olson & Jiang, 2002). In particular, they are consistent with theories that suggest that memory is object based only in that instantiating a new object representation is costly, but that within an object, memory for features is somewhat independent(see Brady, Konkle, & Alvarez, 2011, for review; Fougnie, Asplund, & Marois, 2010; Markov, Tiurina, & Utochkin, 2019; Shin & Ma, 2017; Wang, Cao, Theeuwes, Olivers, & Wang, 2017; Wheeler & Treisman, 2002). Unlike previous work, we showed that feature independence can be found not only for simple stimuli with basic features naturally assumed to be separable (such as color, orientation, or shape), but also for real-world objects whose properties are more complex and meaningfully connected. Therefore, our study suggests that at least partially independent feature storage is a common property of visual working memory representations. It is possible that, under certain conditions, objects could be encoded more holistically (e.g., when visual working memory load is lower), but our results demonstrate that objects are not, by necessity, encoded in a holistic, all-or-none manner.

Because we used considerably more ecologically valid stimuli in these experiments than in previous work, it is important to compare our results with the results reported in the previous literature with simple stimuli. In a study using simple features such as color and orientation (Bays et al., 2009), the percentage of swap errors for set size 4, with similar encoding duration and similar items localization on the screen to the current study, was around 0.11, which is similar to the percentage of swap errors in our experiments (around 0.10). So, the complexity of the features and meaningfulness of remembered stimuli did not make a very large difference in the frequency of binding errors, suggesting that the features of real-world objects are stored with a similar degree of independence to colors and orientations. Note that this study did not aim to discriminate between the visual and semantic features which underlie the state and exemplar discriminations, and therefore which are somewhat independently represented. This complicated question is open for future investigation.

However, these results do provide some evidence for the idea that objects are stored in a more abstract way than just basic visual features. That is, if participants solely stored shape, color, spatial frequency, orientation, and other basic visual features, then—because state and exemplar comparisons inevitably both rely on a combination of these features—memory for the state and exemplar properties would end up looking “bound.” This is because, even if color or orientation or another of the basic visual features was selectively lost, this would impact both state and exemplar comparisons, so the two would be expected to change together for the most part. Because swap errors are about as common for these properties as with simple features like color and orientation, this provides some evidence that people are representing higher-level, perhaps more semantic features of the objects (which can be misbound or independently forgotten, as in color/orientation).

Although our results show that the information about exemplar and state features of real-world objects is not stored in working memory in a totally holistic, all-or-none manner, it does not mean that these features are stored on completely independent “shelves” somewhere in memory. One promising way of thinking about this is that connection between exemplars and states can be a hierarchically linked structure (Balaban et al., 2020; Brady et al., 2011; Fougnie et al., 2013), still leaving a possibility that these links can be incorrect or lost leading to the observed misreports of state–exemplar conjunctions. Compared with long-term memory (Utochkin & Brady, 2020), these misreports are rarer in working memory, suggesting that the feature representations can be linked more strongly in the latter case (Pertzov et al., 2012).

It is important to note that, in our experiments, we used a relatively extended presentation time (3 seconds), which might raise a question whether observers could go beyond the “pure” encoding capacity of visual working memory putting some part of information into long-term memory. Indeed, there is no gold standard in the literature regarding the critical presentation time for pure working memory, with durations ranging from hundreds milliseconds (Alvarez & Cavanagh, 2004; Bays et al., 2009; Luck & Vogel, 1997; Olson & Jiang, 2002) to several seconds (Bays et al., 2009; Bays et al., 2011; Brady et al., 2013; Brady et al., 2016; Fougnie & Alvarez, 2011; Pertzov et al., 2012), and with no strong evidence that encoding time fundamentally changes the relative contribution of different memory systems. However, we do not completely rule out a long-term component in our experiments, especially given the nature of our real-world stimuli, because they are inherently linked to existing knowledge and could allow for the use of newly encoded long-term representations, although because both the studied items and lures are real-world objects, to discriminate them, participants would need display-specific, newly formed long-term memories, no more than a few seconds old; without encoding the particular items in a particular display, they would be a chance in the test, even using long-term memory. As we discussed elsewhere in this article, both visual and semantic features can underlie the exemplar–state discrimination and some of this information could be supported and enhanced by existing knowledge (Curby, Glazek, & Gauthier, 2009; Brady et al., 2016; Schurgin et al., 2018). However, even with the potential support from long-term memory, we still observe around 10% binding errors, which is comparable with the data from other studies using meaningless stimuli and shorter encoding times (e.g., Bays et al., 2009; Emrich & Ferber, 2012).

It is important to note that accuracy in the exemplar task was always higher than the accuracy in the exemplar–state task, even when two exemplars were studied in same states (Experiments 1 and 2), when memory for conjunctions is in fact not required and memory for states is sufficient. One explanation of such results is that, when both exemplars are presented in the same states, it is hard for observers to know which state features they should remember (e.g., if they saw two empty glasses of water it may not be easy to anticipate that the tested state will be fullness [empty glass and full glass] or brokenness [empty glass and broken glass]). In a condition with different states, observers are more likely to realize which states will be tested and which features they should encode. Thus, it is possible that there are even more binding errors, but we could not detect them because the performance in the same states condition is impaired by a failure to anticipate and encode the proper states. However, it also could be thought that the exemplar task was easier than the exemplar–state task, because of the discrimination required at test—that is, that states are simply harder to discriminate than exemplars. It may seem that recognizing an old exemplar against a new exemplar is, by necessity, always easier than recognizing an old state of an exemplar against a new state of the same exemplar. But in fact, two states of the same exemplar often could be more perceptually different (e.g., a sliced and nonsliced instances of the same apple differ by color, texture, shape, presence or absence of pips, etc.) than two different exemplars in the same state (e.g., two different exemplars of apples that could differ only by color), and most previous work has found that observers are about equally good at recognizing old exemplars versus new exemplars and old states of same exemplars versus their new states (Brady et al., 2008).

To test this idea more directly, we performed an additional analysis of similarity using a deep convolutional neural network that has been previously shown to be a useful model of similarity of real-world objects, successfully predicting which foils are more or less difficult to discriminate (Brady & Störmer, 2020). This additional analysis of similarity confirmed that, in fact, there was quite a lot of heterogeneity in the difficulty of both state and exemplar comparisons. Furthermore, this analysis showed that, even when paired states were more dissimilar than paired exemplars, this did not affect the general pattern we observed, suggesting the patterns arise from binding difficulties rather than differences in the stimuli. Therefore, it is not very likely that overall object similarity can explain the superiority of exemplar memory over state memory. From our additional analysis of exemplar–state report accuracy as a function of nontested item states, we can conclude that a fraction of misreported same-state conjunctions (tested objects) can be accounted for by trials where nontested exemplars were shown in different states, perhaps taking up more encoding time/working memory resources.

What kind of representations could underlie the swap errors observed in our experiments? We suggest a few possible scenarios compatible with our data, all consistent with some form of nonholistic, at least partially independent storage: 1) participants might have strong feature memories (when both exemplar and state information are present) and a failure to bind them. The binding failures, in turn, can take the form of false bindings (remembering full mug *A* and/or empty mug *B*, whereas mug *A* was in fact empty and mug *B* was in fact full) or not remembered bindings (remembering seeing both mugs, as well as fullness and emptiness but not remembering which mug was presented full and which one empty). 2) Another possibility is that some of the features could be (independently) forgotten but observers strategically guess these features—that is, participants might not know, at test, whether one of the mugs was full or empty, but remember seeing that mug. For example, the superior performance in the same state condition of the exemplar–state task could be explained by better memory for repeated states (perhaps owing to chunking these states) and worse memory for different states. The trials where observers correctly reported different states, but reported none of the conjunctions correctly could be the result of the strategic guesses (if I do not remember states, I can randomly choose two different ones). This scenario is possible both with and without good exemplar memory. Although our data do not allow us to distinguish between these scenarios and future research is necessary for it, our principal conclusion is that any of these scenarios require state and exemplar memories to be stored or lost at least partially independently. Any of the scenarios is inconsistent with strongly holistic, bound representations (the strong object hypothesis), as such representations should be indifferent to whether objects are shown in same or different states.

Our results add to the picture of how objects are stored and forgotten across memory systems. Previous work has suggested that long-term memory is likely to store quite a lot of independently represented features of real-world objects (Brady et. al., 2013; Utochkin & Brady, 2020). In particular, using the exemplar and exemplar–state tasks for long-term memory, Utochkin and Brady (2020) showed that observers were at chance when reporting state–exemplar conjunctions of objects presented in different states, although they chose two different states for such objects well above chance and their exemplar memory also was good. Importantly, the difference in recognition accuracies between same-state and different-state pairs of studied exemplars was dramatic (0.74 vs. 0.53, respectively). Our current results do not show such a strongly disruptive effect of different-state objects. Neither exemplar–state report accuracy was near chance in that condition, nor was its difference from same-state trials that large. We, therefore, can conclude that visual working memory can provide more boundedness of representations than visual long-term memory—perhaps in part because of representations of color, shape, orientation, and so on, that by necessity provide useful information about both state and exemplar discriminations. At the same time, the fact that some binding failures can occur after a 1-second retention period in working memory is suggestive that part of the failed conjunction representations in long-term memory may arise when these features are consolidated from working memory. It is also consistent with previous demonstrations that object–location bindings are most susceptible to forgetting in working memory (Pertzov et al., 2012). The links between binding errors in visual working memory and visual long-term memory is an interesting subject for future research.

In Experiment 3, we tested an additional hypothesis following from the idea of independence—namely, that manipulating object locations at encoding and retrieval would produce specific updating failures when an observer reports a feature remembered at that location when another feature at this location is changed. Although this experiment allowed a strong replication of the independence pattern in terms of the same–different state manipulations, and location swaps caused additional failures, we found no interaction between the location manipulation and state manipulation in Experiment 3. From these results we concluded that location updating appears more whole object based (consistent with what Dowd & Golomb, 2019 found for simple features).

Several explanations can be considered to account for the finding of independence of features combined with the demonstration of the whole object location updating. First, the object–location binding problem is a separate problem from feature binding (Treisman, 1996), in that objects and locations are processed to some extent via two separate pathways, ventral and dorsal (Haxby, et al., 1991; Mishkin & Ungerleider, 1982; Wilson, O'Scalaidhe & Goldman-Rakic, 1993). Thus, object–location binding is also a separate process from storing objects and locations (Postma & De Haan, 1996; Postma, Kessels, & van Asselen, 2008), so it is possible that location swaps did not influence binding of the features underlying state and exemplar discrimination. According to our results, it is possible that object–location binding could happen after feature binding, which is consistent with object file theories (Hollingworth & Rasmussen, 2010; Kahneman, Treisman, & Gibbs, 1992) and with the general invariance of location tracking (e.g., in multiple object tracking) to feature information (Flombaum, Scholl, & Santos, 2009; Pylyshyn, 2000) Another explanation is discussed by Utochkin and Brady (2020). The interaction between exemplar and state information could be more complicated than the interaction between parallel representations of low-level features (such as color and orientation). Instead, the somewhat independent storage of features supporting exemplar discrimination and state discrimination could result not from a fully parallel organization but from a hierarchical one (Brady, Konkle, & Alrvarez, 2011; Utochkin & Brady, 2020), with exemplar information on a higher level, while access to state information is possible only when exemplar information is not lost (if I do not remember this mug, I also do not remember whether it was full or empty), but not vice versa (I do not remember whether the mug was full or empty, but I remember it was this high yellow mug). Note that this hierarchy does not contradict the idea that these features are not holistic or all-or-none but partially independent: State information still can be forgotten independently from its exemplar or “migrate” to another exemplar in such a model, and objects are not forgotten in an all-or-none way in such a model. This form of hierarchy can potentially explain the results Experiment 3: location swaps could impair exemplar recognition at a new location (because it was always the exemplar whose location has been manipulated at test in Experiment 3), which entails the loss of access to state information. This structure is similar to the one proposed by familiarity/recollection dichotomies in working memory, where item memory is necessary to access context information or other episodic details (e.g., Mickes, Wais, Wixted, 2009). The hierarchical organization of real-world object storage in working memory is an intriguing possibility that needs further investigation.

Overall, in this work we not only show that binding errors occur for real-world objects, but also investigated how working memory updates information about such objects, thus providing new information about how real-world objects are both maintained and updated, the two most critical features of working memory (Baddeley, 1986; Baddeley & Hitch, 1974; Ecker et al., 2010; Nyberg & Eriksson, 2016). We showed that the features underlying two different discrimination tests about real-world objects are somewhat independently represented inside the object representation, as opposed to entirely holistic and all-or-none, but that location updates appear to work at the level of whole object representations rather than impairing links between internal features.

## References

[bib1] Alvarez, G. A., & Cavanagh, P. (2004). The capacity of visual short-term memory is set both by visual information load and by number of objects. *Psychological Science: A Journal of the American Psychological Society/APS,* 15(2), 106–111, 10.1167/2.7.273. 14738517

[bib2] Asp, I. E., Störmer, V. S., & Brady, T. (2019). Greater visual working memory capacity for visually-matched stimuli when they are recognized as meaningful. *Journal of Cognitive Neuroscience,* 11, 1–17*.* 10.1162/jocn_a_01693 34449847

[bib3] Baddeley, A. D., & Hitch, G. J. (1974). Working memory. In G. A. Bower (ed). *The psychology of learning and motivation: advances in research and theory**,* pp. 47–89. New York: Academic Press.

[bib4] Baddeley, A. D. (1986). *Working memory**.* Oxford, UK: Clarendon Press.

[bib5] Baddeley, A. D. (1966). The influence of acoustic and semantic similarity on long-term memory for word sequences. *Quarterly Journal of Experimental Psychology**,* 18(4), 302–309. 10.1080/14640746608400047 5956072

[bib6] Balaban, H., Assaf, D., Arad Meir, M., & Luria, R. (2020). Different features of real-world objects are represented in a dependent manner in long-term memory. *Journal of Experimental Psychology: General,* 149(7), 1275–1293, 10.1037/xge0000716. 31804123

[bib7] Bapat, A. N., Shafer-Skelton, A., Kupitz, C. N., & Golomb, J. D. (2017). Binding object features to locations: Does the “spatial congruency bias” update with object movement? *Attention, Perception, & Psychophysics,* 79(6), 1682–1694, 10.3758/s13414-017-1350-5. PMC5542004 28584957

[bib8] Bays, P. M. (2016). Evaluating and excluding swap errors in analogue tests of working memory. *Scientific Reports,* 6(2015), 1–14, 10.1038/srep19203. 26758902 PMC4725843

[bib9] Bays, P. M., Catalao, R. F. G., & Husain, M. (2009). The precision of visual working memory is set by allocation of a shared resource. *Journal of Vision,* 9(10), 7–7, 10.1167/9.10.7. PMC3118422 19810788

[bib10] Bays, P. M., Wu, E. Y., & Husain, M. (2011). Storage and binding of object features in visual working memory. *Neuropsychologia,* 49(6), 1622–1631, 10.1016/j.neuropsychologia.2010.12.023. 21172364 PMC3119435

[bib11] Brady, T. F., Konkle, T., & Alvarez, G. A. (2011). A review of visual memory capacity: Beyond individual items and toward structured representations. *Journal of Vision,* 11(5), 4–4, 10.1167/11.5.4. PMC3405498 21617025

[bib12] Brady, T. F., Konkle, T., Alvarez, G. A., & Oliva, A. (2008). Visual long-term memory has a massive storage capacity for object details. *Proceedings of the National Academy of Sciences of the United States of America,* 105(38), 14325–14329, 10.1073/pnas.0803390105. 18787113 PMC2533687

[bib13] Brady, T. F., Konkle, T., Alvarez, G. A., & Oliva, A. (2013). Real-world objects are not represented as bound units: Independent forgetting of different object details from visual memory. *Journal of Experimental Psychology. General,* 142(3), 791–808, 10.1037/a0029649. 22905874

[bib14] Brady, T. F., & Störmer, V. S. (2020). Greater capacity for objects than colors in visual working memory: Comparing memory across stimulus spaces requires maximally dissimilar foils. PsyArXiv, 10.31234/osf.io/25t76. 37973770

[bib15] Brady, T. F., & Störmer, V. S. (in press). The role of meaning in visual working memory: Real-world objects, but not simple features, benefit from deeper processing. *Journal of Experimental Psychology: Learning, Memory and Cognition*, doi:10.31234/osf.io/kzvdg. 33764123

[bib16] Brady, T. F., Störmer, V. S., & Alvarez, G. A. (2016). Working memory is not fixed-capacity: More active storage capacity for real-world objects than for simple stimuli. *Proceedings of the National Academy of Sciences of the United States of America,* 113(27), 7459–7464, 10.1073/pnas.1520027113. 27325767 PMC4941470

[bib17] Conway, B. R. (2009). Color vision, cones, and color-coding in the cortex. *Neuroscientist,* 15(3), 274–290, 10.1177/1073858408331369. 19436076

[bib18] Cowan, N. (2001). The magical number 4 in short-term memory: A reconsideration of mental storage capacity. *Behavioral and Brain Sciences,* 24(1), 87–185, 10.1017/S0140525=01003922. 11515286

[bib19] Cowan, N., Chen, Z., & Rouder, J. N. (2004). Constant capacity in an immediate serial-recall task: A logical sequel to Miller (1956). *Psychological Science,* 15(9), 634–640, 10.1111/j.0956-7976.2004.00732.x. 15327636

[bib20] Cowan, N., Blume, C. L., & Saults, J. S. (2013). Attention to attributes and objects in working memory. *Journal of Experimental Psychology: Learning, Memory, and Cognition,* 39(3), 731–747, 10.1037/a0029687. PMC3825193 22905929

[bib21] Curby, K. M., & Gauthier, I. (2007). A visual short-term memory advantage for faces. *Psychonomic Bulletin & Review,* 14(4), 620–628, 10.3758/BF03196811. 17972723

[bib22] Curby, K. M., Glazek, K., & Gauthier, I. (2009). A visual short-term memory advantage for objects of expertise. *Journal of Experimental Psychology: Human Perception and Performance,* 35(1), 94–107, 10.1037/0096-1523.35.1.94. 19170473 PMC4159943

[bib23] Dent, K., & Smyth, M. M. (2005). Verbal coding and the storage of form-position associations in visual–spatial short-term memory. *Acta Psychologica,* 120(2), 113–140, 10.1016/J.ACTPSY.2005.03.004. 15896699

[bib24] DiCarlo, J. J., Zoccolan, D., & Rust, N. C. (2012). How does the brain solve visual object recognition? *Neuron,* 73(3), 415–434, 10.1016/j.neuron.2012.01.010. 22325196 PMC3306444

[bib25] Dowd, E. W., & Golomb, J. D. (2019). Object-feature binding survives dynamic shifts of spatial attention. *Psychological Science,* 30(3), 343–361, 10.1177/0956797618818481. 30694718 PMC6419262

[bib26] Ecker, U. K. H., Lewandowsky, S., Oberauer, K., & Chee, A. E. H. (2010). The components of working memory updating: An experimental decomposition and individual differences. *Journal of Experimental Psychology: Learning, Memory, and Cognition,* 36(1), 170–189, 10.1037/a0017891. 20053053

[bib27] Emrich, S. M., & Ferber, S. (2012). Competition increases binding errors in visual working memory. *Journal of Vision,* 12(4), 12–12, 10.1167/12.4.12. 22523399

[bib28] Erez, J., Cusack, R., Kendall, W., & Barense, M. D. (2016). Conjunctive coding of complex object features. *Cerebral Cortex,* 26(5), 2271–2282, 10.1093/cercor/bhv081. 25921583 PMC4830298

[bib29] Faul, F., Erdfelder, E., Bucher, A., & Lang, A.-G. (2009). G*Power 3: A flexible statistical power analysis program for the social, behavioral, and biomedical sciences. *Behavioral Research Methods,* 39, 175–191, Retrieved from http://www.psycho.uni-duesseldorf.de/abteilungen/aap/gpower3/download-and-register/Dokumente/GPower3-BRM-Paper.pdf. 10.3758/bf03193146 17695343

[bib30] Fiebach, C. J., Rissman, J., & D'Esposito, M. (2006). Modulation of inferotemporal cortex activation during verbal working memory maintenance. *Neuron,* 51(2), 251–261, 10.1016/j.neuron.2006.06.007. 16846859 PMC4544870

[bib31] Flombaum, J. I., Scholl, B. J., & Santos, L. R. (2009). Spatiotemporal priority as a fundamental principle of object persistence. In: Hood, B. M., & Santos, L. R., editors. The *origins of object knowledge*. Oxford, UK: Oxford University Press, 10.1093/acprof:oso/9780199216895.003.0006.

[bib32] Fougnie, D., & Alvarez, G. A. (2011). Object features fail independently in visual working memory: Evidence for a probabilistic feature-store model. *Journal of Vision,* 11(12), 3, 10.1167/11.12.3. PMC3279121 21980189

[bib33] Fougnie, D., Asplund, C. L., & Marois, R. (2010). What are the units of storage in visual working memory? *Journal of Vision,* 10(12), 27, 10.1167/10.12.27. PMC3070178 21047759

[bib34] Fougnie, D., Cormiea, S. M., & Alvarez, G. A. (2013). Object-based benefits without object-based representations. *Journal of Experimental Psychology: General,* 142(3), 621–626, 10.1037/a0030300. 23067063

[bib35] Fougnie, D., & Marois, R. (2009). Attentive tracking disrupts feature binding in visual working memory. *Visual Cognition,* 17(1–2), 48–66, 10.1080/13506280802281337. 19609460 PMC2710819

[bib36] Golomb, J. D., Kupitz, C. N., & Thiemann, C. T. (2014). The influence of object location on identity: A “spatial congruency bias”. *Journal of Experimental Psychology: General,* 143(6), 2262–2278, 10.1037/xge0000017. 25222263

[bib37] Gorgoraptis, N., Catalao, R. F. G., Bays, P. M., & Husain, M. (2011). Dynamic Updating of Working Memory Resources for Visual Objects. *Journal of Neuroscience,* 31(23), 8502–8511, 10.1523/JNEUROSCI.0208-11.2011. 21653854 PMC3124758

[bib38] Harrison, S. A., & Tong, F. (2009). Decoding reveals the contents of visual working memory in early visual areas. *Nature,* 458(7238), 632–635, 10.1038/nature07832. 19225460 PMC2709809

[bib39] Haxby, J. V., Grady, C. L., Horwitz, B., Ungerleider, L. G., Mishkin, M., Carson, R. E., … Rapoport, S. I. (1991). Dissociation of object and spatial visual processing pathways in human extrastriate cortex. *Proceedings of the National Academy of Sciences of the United States of America,* 88(5), 1621–1625, 10.1073/pnas.88.5.1621. 2000370 PMC51076

[bib40] Hollingworth, A., & Rasmussen, I. P. (2010). Binding objects to locations: The relationship between object files and visual working memory. *Journal of Experimental Psychology: Human Perception and Performance,* 36(3), 543–564, 10.1037/a0017836. 20515188 PMC3163079

[bib41] Janini, D., & Konkle, T. (2019). A Pokémon-sized window into the human brain. *Nature Human Behaviour,* 3, 552–553, 10.1038/s41562-019-0594-6. 31061488

[bib42] JASP Team. (2018). *JASP (Version 0.8.5.1) [Computer software]*. Amsterdam, the Netherlands: JASP.

[bib43] Kahneman, D., Treisman, A, & Gibbs, B. J. (1992). The reviewing of object files: Object-specific integration of information. *Cognitive Psychology,* 24(2), 175–219, 10.1016/0010-0285(92)90007-O. 1582172

[bib44] Kaiser, D., Stein, T., & Peelen, M. V. (2015). Real-world spatial regularities affect visual working memory for objects. *Psychonomic Bulletin & Review,* 22(6), 1784–1790, 10.3758/s13423-015-0833-4. 25896215

[bib45] Konkle, T., Brady, T. F., Alvarez, G. A., & Oliva, A. (2010). Conceptual distinctiveness supports detailed visual long-term memory for real-world objects. *Journal of Experimental Psychology: General,* 139(3), 558–578, 10.1037/a0019165. 20677899 PMC3398125

[bib46] Konkle, T., & Oliva, A. (2012). A Real-world size organization of object responses in occipitotemporal cortex. *Neuron,* 74(6), 1114–1124, 10.1016/j.neuron.2012.04.036. 22726840 PMC3391318

[bib47] Lee, D., & Chun, M. M. (2001). What are the units of visual short-term memory, objects or spatial locations? *Perception & Psychophysics,* 63(2), 253–257, 10.3758/BF03194466. 11281100

[bib48] Lew, T. F., & Vul, E. (2015). Ensemble clustering in visual working memory biases location memories and reduces the Weber noise of relative positions. *Journal of Vision,* 15(4), 10, 10.1167/15.4.10. 26360154

[bib49] Li, L., Miller, E. K., & Desimone, R. (1993). The representation of stimulus familiarity in anterior inferior temporal cortex. *Journal of Neurophysiology,* 69(6), 1918–1929, 10.1152/jn.1993.69.6.1918. 8350131

[bib50] Li, X., Xiong, Z., Theeuwes, J., & Wang, B. (2020). Visual memory benefits from prolonged encoding time regardless of stimulus type. *Journal of Experimental psychology. Learning, Memory, and Cognition,* 46(10), 1998–2005. 10.1037/xlm0000847 32437186

[bib51] Long, B., Konkle, T., Cohen, M. A., & Alvarez, G. A. (2016). Mid-level perceptual features distinguish objects of different real-world sizes. *Journal of Experimental Psychology: General,* 145(1), 95–109, 10.1037/xge0000130. 26709591

[bib52] Long, B., Moher, M., Carey, S., & Konkle, T. (2019). Real-world size is automatically encoded in preschoolers’ object representations. *Journal of Experimental Psychology: Human Perception and Performance,* 45(7), 863–876, 10.1037/xhp0000619. 30985176

[bib53] Luck, S. J., & Vogel, E. K. (1997). The capacity of visual working memory for features and conjunctions. *Nature,* 390(6657), 279–281, 10.1038/36846. 9384378

[bib54] Luria, R., & Vogel, E. K. (2011). Shape and color conjunction stimuli are represented as bound objects in visual working memory. *Neuropsychologia,* 49(6), 1632–1639, 10.1016/j.neuropsychologia.2010.11.031. 21145333 PMC3095682

[bib55] Markov, Y. A., Tiurina, N. A., & Utochkin, I. S. (2019). Different features are stored independently in visual working memory but mediated by object-based representations. *Acta Psychologica,* 197, 52–63, 10.1016/j.actpsy.2019.05.003. 31100548

[bib56] Mickes, L., Wais, P. E., & Wixted, J. T. (2009). Recollection is a continuous process: Implications for dual-process theories of recognition memory: Research Article. *Psychological Science,* 20(4), 509–515, 10.1111/j.1467-9280.2009.02324.x. 19320859

[bib57] Miller, G. A. (1956). The magical number seven, plus or minus two: Some limits on our capacity for processing information. *Psychological Review,* 101(2), 343–352, 10.1037/h0043158. 8022966

[bib58] Miller, E. K., Erickson, C. A., & Desimone, R. (1996). Neural mechanisms of visual working memory in prefrontal cortex of the macaque. *Journal of Neuroscience,* 16(16), 5154–5167, 10.1523/JNEUROSCI.16-16-05154.1996. 8756444 PMC6579322

[bib59] Mishkin, M., & Ungerleider, L. G. (1982). Contribution of striate inputs to the visuospatial functions of parieto-preoccipital cortex in monkeys. *Behavioural Brain Research,* 6(1), 57–77, 10.1016/0166-4328(82)90081-X. 7126325

[bib60] Nyberg, L., & Eriksson, J. (2016). Working memory: Maintenance, updating, and the realization of intentions. *Cold Spring Harbor Perspectives in Biology,* 8(2), a021816, 10.1101/cshperspect.a021816. PMC4743080 26637287

[bib61] O'Donnell, R. E., Clement, A., & Brockmole, J. R. (2018). Semantic and functional relationships among objects increase the capacity of visual working memory. *Journal of Experimental Psychology: Learning Memory and Cognition,* 44(7), 1151–1158, 10.1037/xlm0000508. 29648871

[bib62] Oberauer, K., & Lin, H. (2017). An interference model of visual working memory. *Psychological Review,* 124(1), 1–39, 10.1037/rev0000044. 27869455

[bib63] Olson, I. R., & Jiang, Y. (2002). Is visual short-term memory object based? Rejection of the “strong-object” hypothesis. *Perception and Psychophysics,* 64(7), 1055–1067, 10.3758/BF03194756. 12489661

[bib64] Paik, S. B., & Ringach, D. L. (2011). Retinal origin of orientation maps in visual cortex. *Nature Neuroscience,* 14(7), 919–925, 10.1038/nn.2824. 21623365 PMC3196663

[bib65] Park, Y. E., Sy, J. L., Hong, S. W., & Tong, F. (2017). Reprioritization of features of multidimensional objects stored in visual working memory. *Psychological Science,* 28(12), 1773–1785. 28957016 10.1177/0956797617719949 PMC5725244

[bib66] Peirce, J., Gray, J. R., Simpson, S., MacAskill, M., Höchenberger, R., Sogo, H., … Lindeløv, J. K. (2019). PsychoPy2: Experiments in behavior made easy. *Behavior Research Methods,* 51(1), 195–203, 10.3758/s13428-018-01193-y. 30734206 PMC6420413

[bib67] Pertzov, Y., Dong, M. Y., Peich, M. C., & Husain, M. (2012). Forgetting what was where: The fragility of object-location binding. *PLoS One,* 7(10), e48214, 10.1371/journal.pone.0048214. 23118956 PMC3485137

[bib68] Postma, A., & De Haan, E. H. F. (1996). What Was Where? Memory for Object Locations. *Quarterly Journal of Experimental Psychology Section A,* 49(1), 178–199, 10.1080/713755605. 8920102

[bib69] Postma, A., Kessels, R. P. C., & van Asselen, M. (2008). How the brain remembers and forgets where things are: The neurocognition of object-location memory. *Neuroscience and Biobehavioral Reviews,* 32(8), 1339–1345, 10.1016/j.neubiorev.2008.05.001. 18562002

[bib70] Pylyshyn, Z. W. (2000). Situating vision in the world. *Trends in Cognitive Sciences,* 4(5), 197–207, 10.1016/S1364-6613(00)01477-7. 10782106

[bib71] Quirk, C., Adam, K. C. S., & Vogel, E. K. (2020). No evidence for an object working memory capacity benefit with extended viewing time. *Eneuro,* 7(5), ENEURO.0150–20.2020, 10.1523/ENEURO.0150-20.2020. PMC7519167 32859722

[bib72] Ranganath, C., DeGutis, J., & D'Esposito, M. (2004). Category-specific modulation of inferior temporal activity during working memory encoding and maintenance. *Cognitive Brain Research,* 20(1), 37–45, 10.1016/j.cogbrainres.2003.11.017. 15130587

[bib73] Rouder, J. N., Speckman, P. L., Sun, D., Morey, R. D., & Iverson, G. (2009). Bayesian t tests for accepting and rejecting the null hypothesis. *Psychonomic Bulletin & Review,* 16(2), 225–237, 10.3758/PBR.16.2.225. 19293088

[bib74] Serences, J. T., Ester, E. F., Vogel, E. K., & Awh, E. (2009). Stimulus-specific delay activity in human primary visual cortex. *Psychological Science,* 20(2), 207–214, 10.1111/j.1467-9280.2009.02276.x. 19170936 PMC2875116

[bib75] Serences, J. (2016). Neural mechanisms of information storage in visual short-term memory. *Vision Research**,* 128, 53–67, 10.1016/j.visres.2016.09.010.. 27668990 PMC5079778

[bib76] Shafer-Skelton, A., Kupitz, C. N., & Golomb, J. D. (2017). Object-location binding across a saccade: A retinotopic spatial congruency bias. *Attention, Perception, & Psychophysics,* 79(3), 765–781, 10.3758/s13414-016-1263-8. PMC5354979 28070793

[bib77] Shin, H., & Ma, W. J. (2017). Visual short-term memory for oriented, colored objects. *Journal of Vision,* 17(9), 1–19, 10.1167/17.9.12. PMC5558900 28813568

[bib78] Simonyan, K., & Zisserman, A. (2014). Very deep convolutional networks for large-scale image recognition. arXiv,1409.1556

[bib79] Schurgin, M. W., Cunningham, C. A., Egeth, H. E., & Brady, T. F. (2018). Visual long-term memory can replace active maintenance in visual working memory. *BioRxiv*, 381848, 10.1101/381848.

[bib80] Spachtholz, P., & Kuhbandner, C. (2017). Visual long-term memory is not unitary: Flexible storage of visual information as features or objects as a function of affect. *Cognitive, Affective and Behavioral Neuroscience,* 17, 1141–1150, 10.3758/s13415-017-0538-4. 28924746

[bib81] Treisman, A. (1996). The binding problem. *Current Opinion in Neurobiology,* 6(2), 171–178, 10.1016/S0959-4388(96)80070-5. 8725958

[bib82] Treisman, A. (1999). Solutions to the binding problem: Progress through controversy and convergence. *Neuron,* 24(1), 105–110, 10.1016/S0896-6273(00)80826-0. 10677031

[bib83] Treisman, A. (2006). How the deployment of attention determines what we see. *Visual Cognition,* 14, 411–443, 10.1080/13506280500195250. 17387378 PMC1832144

[bib84] Utochkin, I. S., & Brady, T. F. (2020). Independent storage of different features of real-world objects in long-term memory. *Journal of Experimental Psychology: General,* 149(3), 530–549, 10.1037/xge0000664. 31414858

[bib85] van den Honert, R. N., McCarthy, G., & Johnson, M. K. (2017). Holistic versus feature-based binding in the medial temporal lobe. *Cortex,* 91, 56–66, 10.1016/j.cortex.2017.01.011. 28215821 PMC5446797

[bib86] van Lamsweerde, A. E., Beck, M. R., & Elliott, E. M. (2015). Retrieval from long-term memory reduces working memory representations for visual features and their bindings. *Memory & Cognition,* 43, 237–246, doi:10.3758/s13421-014-0468-0. 25301564

[bib87] Vogel, E. K., Woodman, G. F., & Luck, S. J. (2001). Storage of features, conjunctions and objects in visual working memory. *Journal of Experimental Psychology. Human Perception and Performance,* 27(1), 92–114, 10.1037//0096-1523.27.1.92. 11248943

[bib88] Wagenmakers, E.-J., Love, J., Marsman, M., Jamil, T., Ly, A., Verhagen, J., . . . Morey, R. D. (2017). Bayesian inference for psychology. Part II: Example applications with JASP. *Psychonomic Bulletin & Review**,* 25, 58–76, 10.3758/s13423-017-1323-7. PMC5862926 28685272

[bib89] Wang, B., Cao, X., Theeuwes, J., Olivers, C. N. L., & Wang, Z. (2017). Separate capacities for storing different features in visual working memory. *Journal of Experimental Psychology: Learning, Memory, and Cognition,* 43(2), 226–236, 10.1037/xlm0000295. 27399920

[bib90] Wheeler, M. E., & Treisman, A. M. (2002). Binding in short-term visual memory. *Journal of Experimental Psychology. General,* 131(1), 48–64, 10.1037//0096-3445.131.1.48. 11900102

[bib91] Wilson, F. A. W., Scalaidhe, S. P., & Goldman-Rakic, P. S. (1993). Dissociation of object and spatial processing domains in primate prefrontal cortex. *Science,* 260(5116), 1955–1958, 10.1126/science.8316836. 8316836

[bib92] Xie, W., & Zhang, W. (2017). Familiarity increases the number of remembered Pokémon in visual short-term memory. *Memory & Cognition,* 45(4), 677–689, 10.3758/s13421-016-0679-7. 27933560

[bib93] Xu, Y. (2002). Encoding color and shape from different parts of an object in visual short-term memory. *Perception & Psychophysics,* 64(8), 1260–1280, 10.3758/BF03194770. 12519024

[bib94] Xu, Y., & Chun, M. M. (2006). Dissociable neural mechanisms supporting visual short-term memory for objects. *Nature,* 440(7080), 91–95, 10.1038/nature04262. 16382240

[bib95] Xu, Y. (2017). Reevaluating the sensory account of visual working memory storage. *Trends in Cognitive Sciences**,* 21(10), 794–815. 28774684 10.1016/j.tics.2017.06.013

